# Potential of Polar Lipids Isolated from the Marine Sponge *Haliclona* (*Halichoclona*) *vansoesti* against Melanoma

**DOI:** 10.3390/ijms25137418

**Published:** 2024-07-06

**Authors:** Nadia Ruocco, Genoveffa Nuzzo, Serena Federico, Roberta Esposito, Carmela Gallo, Marcello Ziaco, Emiliano Manzo, Angelo Fontana, Marco Bertolino, Giacomo Zagami, Valerio Zupo, Clementina Sansone, Maria Costantini

**Affiliations:** 1 Department of Ecosustainable Marine Biotechnology, Stazione Zoologica Anton Dohrn, Calabria Marine Centre, C. da Torre Spaccata, 87071 Amendolara, Italy; nadia.ruocco@szn.it; 2 Bio-Organic Chemistry Unit, Institute of Biomolecular Chemistry CNR, Via Campi Flegrei 34, 80078 Pozzuoli, Italy; nuzzo.genoveffa@icb.cnr.it (G.N.); carmen.gallo@icb.cnr.it (C.G.); m.ziaco@icb.cnr.it (M.Z.); afontana@icb.cnr.it (A.F.); 3 Department of Ecosustainable Marine Biotechnology, Stazione Zoologica Anton Dohrn, Via Ammiraglio Ferdinando Acton, 55, 80133 Napoli, Italy; serena.federico@szn.it (S.F.); clementina.sansone@szn.it (C.S.); 4 Department of Earth, Environmental and Life Sciences, University of Genoa, Corso Europa 26, 16132 Genova, Italy; 5 Laboratory of Bio-Organic Chemistry and Chemical Biology, Dipartimento di Biologia, Università di Napoli “Federico II”, Via Cupa Nuova Cinthia 21, 80126 Napoli, Italy; 6 Department of Biological, Chemical, Pharmaceutical and Environmental Sciences, University of Messina, 98100 Messina, Italy; 7 Department of Ecosustainable Marine Biotechnology, Stazione Zoologica Anton Dohrn, Ischia Marine Center, 80077 Ischia, Italy; vzupo@szn.it

**Keywords:** SPE fractionation, marine biotechnology, melanoma, sphingoid lipids, porifera

## Abstract

Marine sponges represent a good source of natural metabolites for biotechnological applications in the pharmacological, cosmeceutical, and nutraceutical fields. In the present work, we analyzed the biotechnological potential of the alien species *Haliclona* (*Halichoclona*) *vansoesti* de Weerdt, de Kluijver & Gomez, 1999, previously collected in the Mediterranean Sea (Faro Lake, Sicily). The bioactivity and chemical content of this species has never been investigated, and information in the literature on its Caribbean counterpart is scarce. We show that an enriched extract of *H. vansoesti* induced cell death in human melanoma cells with an IC_50_ value of 36.36 µg mL^−1^, by (i) triggering a pro-inflammatory response, (ii) activating extrinsic apoptosis mediated by tumor necrosis factor receptors triggering the mitochondrial apoptosis via the involvement of Bcl-2 proteins and caspase 9, and (iii) inducing a significant reduction in several proteins promoting human angiogenesis. Through orthogonal SPE fractionations, we identified two active sphingoid-based lipid classes, also characterized by nuclear magnetic resonance and mass spectrometry, as the main components of two active fractions. Overall, our findings provide the first evaluation of the anti-cancer potential of polar lipids isolated from the marine sponge *H.* (*Halichoclona*) *vansoesti*, which may lead to new lead compounds with biotechnological applications in the pharmaceutical field.

## 1. Introduction

The discovery of new anti-cancer metabolites from marine organisms is continuously occurring, with some successful examples of commercial drugs currently being used in clinical practices [1,2,3]. Marine sponges play a key role in producing bioactive molecules [4,5,6].

The anti-cancer activity of marine sponge-derived compounds has been the most studied, with the first drug approved in 1969 for the treatment of leukemia [7]. So far, different approaches have been applied to discover novel anti-tumor drugs from sponges and/or their symbionts, requiring classical preliminary screening on human cancer cell lines often coupled to the purification and structural characterization of chemical compounds (e.g., nucleosides, macrolides, and sterols) exhibiting such activities [8,9,10,11,12,13,14].

Melanoma is a cutaneous cancer spread worldwide, with an increasing incidence, particularly in white populations. Statistical data have reported that its early diagnosis is extremely important to prevent metastasis and to promote a high 5-year survival rate [15]. Several therapies have been developed to treat melanoma, including classical chemotherapy with anti-proliferative drugs and immunotherapy targeting specific mediators of inflammation [16]. Recently, biomarkers associated with the cell proliferation of metastatic melanoma, such as mutated genes involved into key signaling pathways, have also been discovered. Targeted therapies based on inhibitors of these aberrant genes favoring malignant phenotypes have already been approved by the Food and Drug Administration (FDA), whereas others are still in clinical trials [17]. Current chemotherapies have been testing the induction on cancer cells of immunogenic cell death (ICD) alone and in combination with immunotherapy [18]. ICD has its beginning with the chronic release starting from dying cells of damage-associated molecular patterns (DAMPs) with the recruitment of several immune factors within the tumor microenvironment (TME) that promote an immune-mediated death of cancer cells. Specifically, DAMPs provoke the maturation of dendritic cells (DCs) that produce pro-inflammatory cytokines activating tumor-specific T lymphocytes [19]. Therefore, the elicitation of T cell-driven immune responses specific to cancer antigens has been found to be a potent method to treat and block tumor development [18]. Recently, applying a bioassay-guided screening for immunomodulatory compounds, has led to the identification of lepadin A, an alkaloid immunogenic cell death-inducing compound [20]. 

The potential pharmacological activity of cerebroside and sphingoid lipids are already reported in the literature [21,22,23,24,25] Glycosphingolipids include amphipathic molecules formed by a long-chain amino alcohol, the sphingoid base, a fatty acid residue linked in turn to its amino group (the ceramide), and a carbohydrate chain linked to the primary hydroxyl group of the ceramide. Cerebrosides are neutral monohexosylceramides with the sugar residue composed by glucose or galactose. In the marine environment, many sphingolipids were mainly isolated from echinoderms and sponges, showing several biotechnological applications in pharmacological fields, such as antitumor, immunomodulatory, and neuritogenic activities [22]. One of the first cerebroside mixture was isolated from the marine sponge *Chondrilla nucula* Schmidt, 1862 [26]. Interestingly, Agelasphin-7a, a-galactocerebrosides isolated from the sponge *Agelas mauritiana* Carter, 1883, showed a potent antitumor activity, leading to the development of the synthetic analogue KRN7000, which acts through stimulation of the immune system [27,28]. Recently, several previously unknown *D*-glucopyranosyl-ceramides were identified from the eastern deep-sea glass sponge, *Aulosaccus* sp. [29,30]. Also, cerebrosides from different marine sources and terrestrial environments were discovered to be a source of biomolecules with pharmacological applications. However, no further in-depth investigations at the genetic level related to treatment with cerebrosides have been reported so far.

Similarly, we tested the methanol extract and related enriched fractions obtained by solid phase extraction (SPE) [3,31] of the marine Demospongiae *Haliclona* (*Halichoclona*) *vansoesti* de Weerdt, de Kluijver & Gomez, 1999 on the human melanoma epithelial cell line A2058. This sponge species, belonging to the Demospongiae class, is native to the Caribbean. We previously reported *H.* (*Halichoclona*) *vansoesti* as an alien species from the Mediterranean Sea, being collected from a meromictic basin (Faro Lake) located in the Strait of Messina [32]. We hypothesized that the presence of this sponge could be the result of global warming, and more generally of global changes, which are strongly affecting the marine environment. At present, to our knowledge, the bioactivity of this sponge species has been poorly investigated, with a total lack of studies focusing on its anti-cancer potential [33,34]. In fact, the only data on metabolites isolated from this sponge, dating back to the last ten years, focuses on (2R, 3R, 7Z)-2-aminotetradec-7-ene-1, 3-diol, a potent antimicrobial metabolite [33], and (2R,3R,7Z)-2-aminotetradec-7-ene-1,3-diol, a new amino alcohol [34]. Overall, this study aimed to detect the potential biotechnological role of metabolites from *H.* (*Halichoclona*) *vansoesti*, thus providing the first evaluation of the anti-cancer potential of polar lipids isolated from this marine sponge species. 

## 2. Results

### 2.1. Bioassay-Guided Fractionation and Chemical Analysis of the Active Fractions

**Figure 1:**
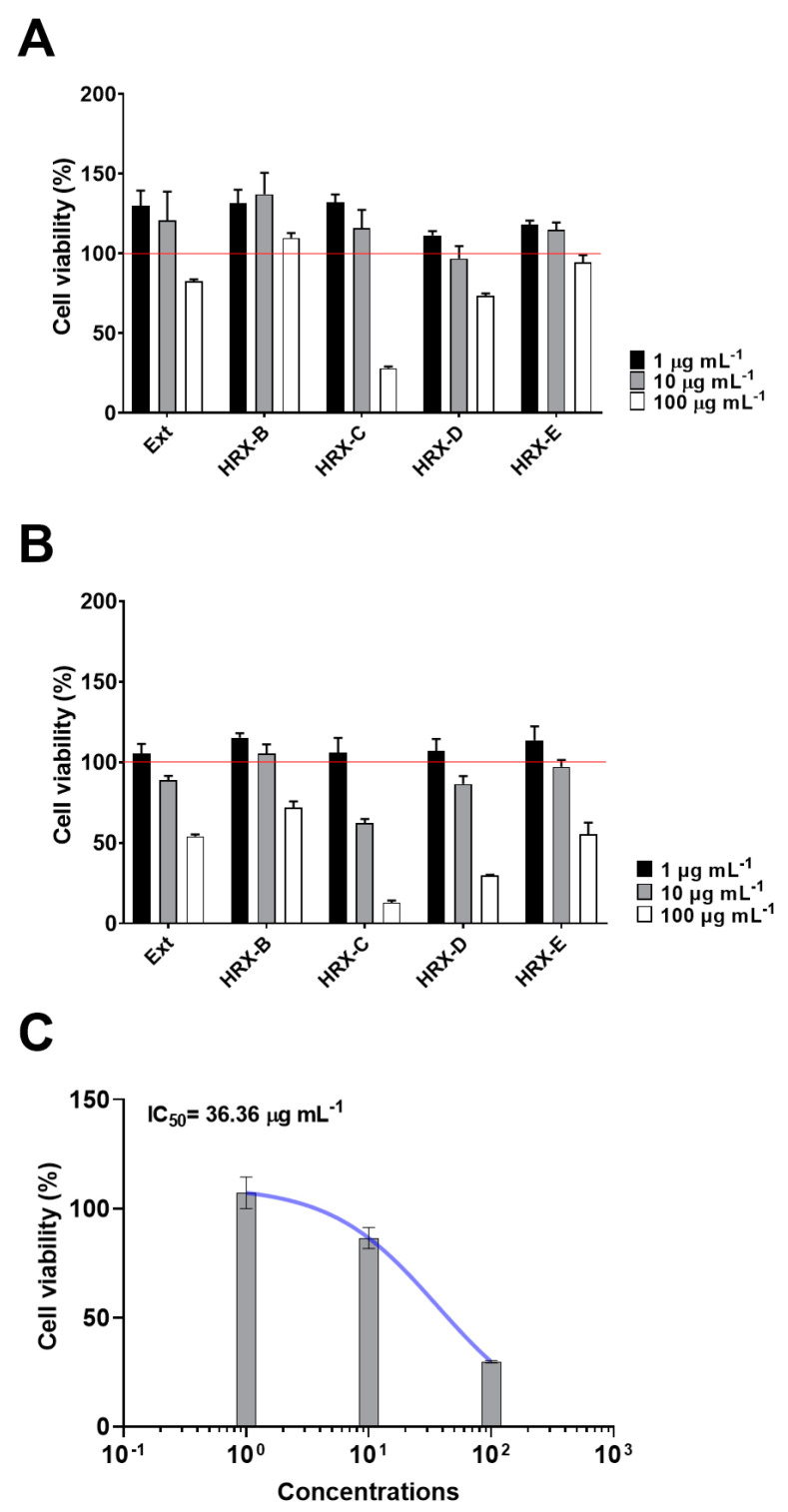
MTT assay of marine sponge extract and fractions on human melanoma cell lines. (**A**) Percentage cell viability of PNT2 cells treated with total extract (Ext) and HRX-B, C, D, and E fractions from *H. vansoesti* at concentrations of 1, 10, and 100 µg mL^−1^. Red line refers to cell viability of 100%. (**B**) Percentage cell viability of A2058 cancer cells treated with total extract (Ext) and HRX-B, C, D, and E fractions from *H. vansoesti* at concentrations of 1, 10, and 100 µg mL^−1^. (**C**) IC_50_ of HRX-D fraction.

Sponge extract and related enriched fractions, obtained by HRX-SPE [3], were analyzed at three different concentrations (1, 10, and 100 µg/mL^−1^) to evaluate the anti-proliferative activity against the A2058 cancer cell line using PNT2 as the somatic cell control. As shown in Figure 1, the results indicated that the HRX fractions C and D were the most cytotoxic. However, due to the strong cytotoxic effect of sample C against normal human PNT2 cells at 100 µg mL^−1^ (Figure 1A), fraction D was selected for subsequent experiments. A specific and dose-dependent cytotoxic effect was identified in the HRX-D fraction on A2058 cells with a low percentage viable cell value (~30%) at 100 µg mL^−1^ (*p* < 0.001) (Figure 1B) and an IC_50_ value of 36.36 µg mL^−1^ (Figure 1C). 

**Figure 2:**
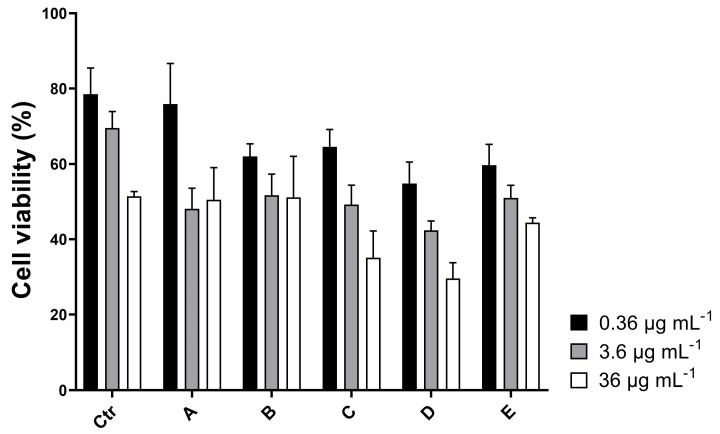
MTT assay of the HILIC fractions (A–E) and sample HRX-D (Ctr) on the human melanoma cell line A2058. Cell viability at IC_50_ concentration (36 µg mL^−1^) and two ten-fold dilutions (3.6 and 0.36 µg mL^−1^, respectively) was reported as a percentage.

**Figure 3:**
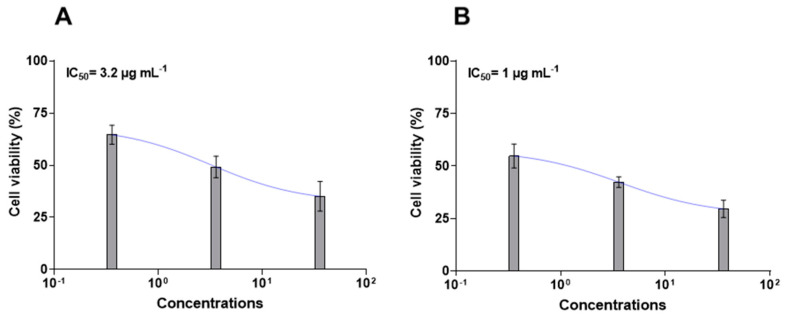
IC_50_ of pro-apoptotic HILIC fractions C (**A**) and D (**B**).

The preliminary ^1^H NMR spectrum (Figure S1) of fraction D indicated that the sample was a mixture of metabolites. Further purification of the active sample with an SPE method based on hydrophilic resin (HILIC-SPE) [20] allowed the separation of different families of lipids, distinctly visible on TLC (Figure S2). All the new fractions obtained by HILIC fractionation of sample HRX-D were then tested on A2058 cells at the IC_50_ concentration and two serial dilutions (ten-fold) to deeply investigate their anti-proliferative activity (Figure 2). The new HILIC samples C and D were found to be the most pro-apoptotic on melanoma cells, with a reduction in cell viability (~55–65%) already visible at the lowest concentration, and IC_50_ values of 3.2 and 1 µg mL^−1^, respectively (Figure 3).

NMR and MS data of the two active fractions clearly indicated the presence of a different families of compounds (Figures S4–S15). 

LC-MS-MS/MS analysis of fraction C suggested the presence of a mixture of cerebrosides differing in the degree of saturation, oxidation, and/or the length of the acyl chain. The main compounds identified as [M-H]^−^ and [M+Cl]^−^ ion peaks are listed in Table 1. Although the NMR spectra of the mixture appeared complex, the characteristics resonances of a sugar, like the anomeric proton at δ_H_ 4.31 (^1^H, d, 7.7 Hz)–δ_C_ 104.3, the diagnostic signal of methine attached to nitrogen at δ_H_ 4.02–δ_C_ 54.4 of the sphingolipid skeleton, several oxygenated methylenes between 3.20 and 4.50 ppm, two oxygenated methylenes at δ_H_ 4.08/3.83 and 3.90/3.70, a long methylene chain protons of fatty acids at δ_H_ 1.30, and the terminal methyl groups at δ_H_ 0.90, were distinguishable (Table 2). 

**Figure 4:**
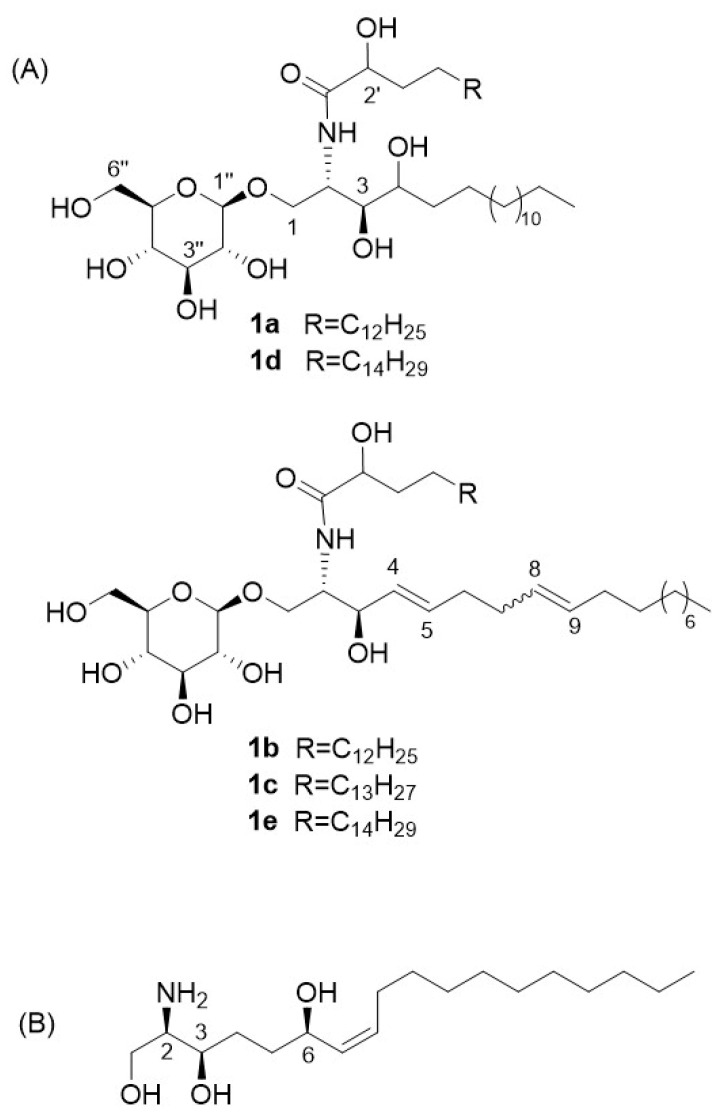
Chemical structure of main compounds related to bioactive fractions C (**A**) and D (**B**).

Using 2D NMR experiments and a comparison with the literature data, it was possible to assign the sugar moiety to a α-glucopyranose (resonances between δ_H_ 4.31 and δ_H_ 3.70 for ^1^H, and δ_C_ 104.1 and δ_C_ 62.5 for ^13^C) [35,36] and the sphingoid base (Table 1), confirming our hypothesis of glucoceramides (Figure 4A). Chemical shift and coupling constant analysis of the anomeric proton (δ_H_ 4.31, d *J* = 7.7 Hz/δ_C_ 104.1) suggested β-orientation of the sugar unit [36,37]. However, COSY and TOCSY correlations supported the presence of two different spin systems from carbon C1 to C6 with a common 2-amino-1,3-dioxygenated moiety (**1a**–**e**). Indeed, the ^1^H and ^13^C NMR signals at position 2 of **1a** and **1d** resonated at 4.28 and 51.5 ppm, while they were shifted at 4.02 and 54.5 ppm in compounds **1b**, **1c**, and **1e**. Both protons showed COSY correlations with a methylene at C-1 and a methine at C-3 oxygenates.

Detailed studies of the fragmentation pattern (Figures S3–S10) allowed us to infer the analogues differing for the hydroxy group in position 4 (**1a**, **1d**) or the Δ^4–5^ double bond of sphingosine (**1b**, **1c**, **1e**). 

Most of the fragmentations in our compounds (Figure S3) were consistent with those previously described by Santalova et al. [30,38]. In ESI^−^ mode, the MS/MS profile of **1a**–**e** showed the diagnostic ion peak of the sugar (C_1_ = *m*/*z* 179.06; B_1_ = *m*/*z* 161.04; A_1_ = *m*/*z* 89.02) and the corresponding loss of the neutral fragment arising from the break between the oxygen and the anomeric carbon [M-H-Glc]^−^ (Y_0_; Figure S10, Table 2). The assignment of the acyl chain is mainly based on the fragmentation in α to the carbonyl moiety (W-2H; Figures S3 and S10). 

Regarding the configuration of the ceramide backbone, the *erythro* stereochemistry at C-2 and C-3 was supported by the agreement of the chemical shifts of the corresponding protons (54.4 and 72.4 ppm, respectively) with the literature data [2,3]. Analogously, the geometry of the double bond between C-4 and C-5 was assigned as *E* by the large value (15.0 Hz) of the coupling constant (Table 2). 

NMR and MS analysis of HILIC fraction D instead indicated the presence of another class of sphingoid base lipids, namely halisphingosines, already reported in various species of the marine sponge genus *Haliclona* [4,5]. The main product, halisphingosine A, is shown in Figure 4B. The NMR data (Table 3, Figures S11–S15) of D displayed the diagnostic signals of a sphingosine-type structure: a proton at δ_H_ 2.96 correlated to a carbon signal at 58.4 due to the methine linked to the amino group, a methylene oxygenated proton at δ_H_ 3.74 and 3.62 (δ_C_ 61.8), a methine oxygenated at δ_H_ 3.66, and a hydroxyl allylic proton at δ_H_ 4.39. 

Although some NMR signals are not well resolved, due to the presence of minor analogues, the cis geometry of the double bond was clearly confirmed through the characteristic coupling constant (*J* = 10.0 Hz) and the carbon chemical shift of CH_2_-9 at 28.6, whereas the stereochemistry of the chiral carbon at C-2, C-3 and C-6 was suggested to be the same as the known metabolite halishingosine A [39] given the similarity of the NMR data with the literature. 

HR-ESI^+^ MS analysis of HILIC fraction D (Figure S14) showed the main peaks at *m*/*z* 316.2844, 297.2740 and 280.2633 related to the [M+H]^+^, [M-H_2_O+H]^+^, [M-2H_2_O+H]^+^ ion peaks of halisphingosine A, respectively. However, additional signals are also present at *m*/*z* 360.3108, 362.3261, 384.3105, 386.3262, 408.3105 and 410.3262, which corresponds well with the following molecular formula C_20_H_42_NO_4_^+^, C_20_H_44_NO_4_^+^, C_22_H_42_NO_4_^+^, C_20_H_44_NO_4_^+^, C_24_H_42_NO_4_^+^, C_24_H_44_NO_4_^+^ (as [M+H]^+^), suggesting the presence of new minor related compounds. The study of MSMS fragments (Figure S15) confirmed the correlation with the main product of these minor analogues, which mainly differed in chain length and degree of unsaturation, and with halisphingosine A for the presence also of one more hydroxyl group in the chain as indicated by the loss of one more water molecule in the pattern of fragmentation. 

### 2.2. Chemical Purification and Characterization of HILIC Fraction C

Chromatographic fractionation of the HILIC fraction C on silica gel column by an elution gradient from 7 to 15% of methanol in dichloromethane yielded two isolated pools of products containing sphingosine with a Δ^4–5^ double bond (**1b**, **1c**, **1e**) or hydroxy group in position 4 (**1a**, **1d**). The purification of these products allowed us to suggest the additional double bond position of the sphingosine chain reported in **1b**, **1c**, and **1e**. In detail, spectroscopic data (Figures S17–S19) indicated the presence of two methylenes at δ_H_ 2.10 and 2.11 coupling with the olefinic protons at δ 5.77 and 5.45 respectively. The olefinic protons at 8,9 resonated at the same chemical shift (5.45 ppm); thus, the geometry of the double bond was not assigned because we do no not have enough data. The NMR and MS data of these two sphingosine families are reported in the Supplementary Material (Figures S16–S22). ESI-MS (negative ions) spectra (Figure S22) counted for a series of peaks between *m*/*z* 712 and 848, suggesting the presence of different fatty acids linked to the sphingosine chain, in addition to **1a**–**e** (Figure 4). However, due to the very limited amount of product available, a separation of the analogues by HPLC was not attempted.

### 2.3. PCR Array

**Figure 5:**
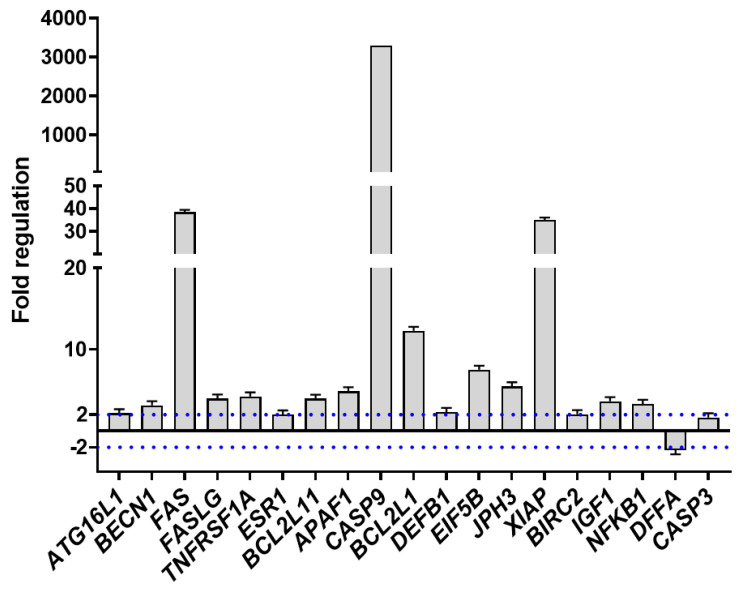
PCR array on A2058 cells exposed to HILIC fraction D. Fold regulation of nineteen apoptotic genes in A2058 cells. Values greater than ±2 and *p* < 0.05 were considered significant (see blue dotted lines).

Gene expression analysis on A2058 cells treated with HILIC samples C and D at IC_50_ concentrations (3.2 and 1 µg mL^−1^, respectively) revealed several molecular targets that were significantly activated (*p* > 0.05) by both subfractions in comparison to the control (see Tables S1–S4). In the case of sample C, different biological pathways were simultaneously triggered after 2 h of treatment. In fact, specific genes for the autophagic cascade, such as *autophagy-related 12 homolog* (*ATG12*, 4.78 fold regulation), *autophagy-related 16-like 1* (*ATG16L1*, 12.64 fold regulation), *autophagy-related 5 homolog* (*ATG5*, 11.75 fold regulation), *autophagy-related 7 homolog* (*ATG7*, 25.8 fold), and *beclin 1* (*BECN1*, 6.67 fold regulation), were significantly upregulated (see Table S1 for the genes and fold change values). Moreover, *B-cell leukemia/lymphoma 2* (*BCL2*, 40.34-fold regulation), *BCL2-like 1* (*BCL2L1*, 37.22-fold regulation), *apoptotic peptidase activating factor 1* (*APAF-1*, 23.85-fold regulation), *caspase 1* (*CASP1*, 20.2-fold regulation), *caspase 2* (*CASP2*, 13.48-fold regulation), *caspase 3* (*CASP3*, 21.23-fold regulation), *caspase 6* (*CASP6*, 25.11-fold regulation), *caspase 7* (*CASP7*, 84.01-fold regulation), and *caspase 9* (*CASP9*, 132.84-fold regulation), plus *tumor necrosis factor* (*TNF*, 19.83-fold regulation), *tumor necrosis factor receptor superfamily*, *member 10a* (*TNFRSF10A*, 35403.22-fold regulation), *tumor necrosis factor receptor superfamily*, *member 11b* (*TNFRSF11B*, 25.11-fold regulation), and *tumor necrosis factor receptor superfamily*, *member 1A* (*TNFRSF1A*, 36.82-fold regulation) increased their expression, revealing the presence of different apoptotic events in A2058 cells. On the contrary, HILIC fraction D induced a selected molecular pathway by upregulating some gene factors involved in autophagy and in both extrinsic and mitochondrial apoptosis (Figure 5).

For instance, *ATG16L1* (2.16-fold regulation) and *BECN1* (3.14-fold regulation), involved in the autophagic events were upregulated. Moreover, *TNF receptor superfamily*, *member 6* (*FAS*, 38.45-fold regulation) and its ligand *FASLG* (3.97-fold regulation), *TNFRSF1A* (4.24-fold regulation), *estrogen receptor 1* (*ESR1*, 2.02-fold regulation), *CASP9* (3293.15-fold regulation), *BCL-2-like 11 apoptosis facilitator* (*BCL2L11* or *BIM*, 3.94-fold regulation), and *APAF-1* (4.86-fold regulation) significantly increased their expression, together with some defensive and inhibitor genes, such as *BCL-2-like 1* (12.3-fold regulation), *eukaryotic translation initiation factor 5B* (*EIF5B*, 7.51-fold regulation), *X-linked inhibitor of apoptosis* (*XIAP*, 35-fold regulation), *baculoviral IAP repeat containing 2* (*BIRC2*, 2.05-fold regulation), *insulin-like growth factor 1* (*IGF1*, 3.64-fold regulation), and *nuclear factor of kappa light polypeptide gene enhancer in B-cells 1* (*NFKB1*, 3.32-fold regulation). Interestingly, the sponge fraction also downregulated *DNA fragmentation factor*, *alpha polypeptide* (*DFFA*, −2.36-fold regulation) which is an apoptotic regulator triggering DNA fragmentation, and did not switch the effector caspase *CASP3* on or off(1.66-fold regulation). 

### 2.4. ImmunoArray

**Figure 6:**
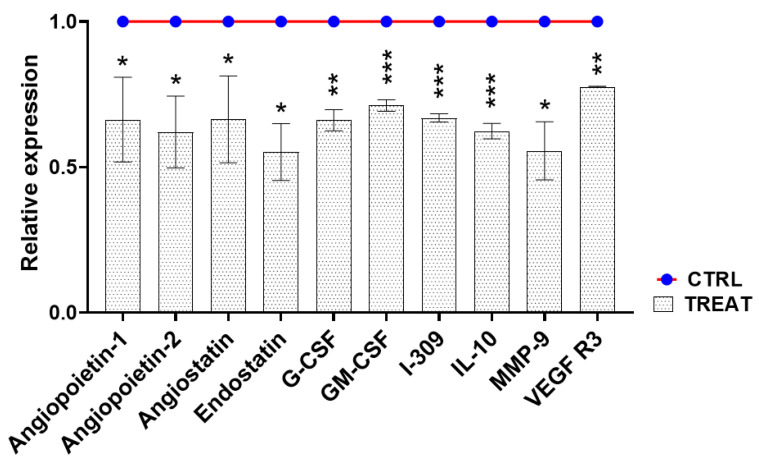
ImmunoArray on A2058 cells treated with the HILIC fraction D. Relative expression of ten proteins involved in human angiogenesis in treated A2058 cells normalized vs. control (untreated cells). A reduction of >30% was considered significant. * *p* value < 0.05; ** *p* value < 0.01; *** *p* value < 0.001.

Immunogenic cell death was then validated through ImmunoArray analysis by focusing on fraction D, denoting an inhibition of proangiogenic factor secretion in culture medium (Figure 6 and Figure S24). 

In particular, the expression and release of several proteins involved in human angiogenesis were evaluated in treated human melanoma cells in comparison to controls. Of the proteins analyzed, ten were significantly depleted (at least a 30% reduction) in A2058 cells after 24 h of treatment with HILIC fraction D at IC_50_ concentration (1 µg mL^−1^). 

In detail, a considerable reduction (~40%, *p* < 0.05) in angiopoietin-1 and angiopoietin-2, was measured. ImmunoArray detection also indicated a relevant decline in granulocyte colony-stimulating factor (G-CSF, ~30%, *p* < 0.01) and granulocyte-macrophage colony-stimulating factor (GM-CSF, ~30%, *p* < 0.001). Moreover, chemokine I-309, significantly decreased, with a percentage of about 30% with respect to control samples (*p* < 0.001). Vascular endothelial growth factor receptor 3 (VEGF R3) and metalloproteinase 9 (MMP-9) also reduced their expression in response to the sponge fraction in melanoma cells by about 50% (*p* < 0.05) and 30% (*p* < 0.01), respectively. Interestingly, the expression of angiostatin, endostatin, and interleukin-10 (IL-10) significantly decreased (~40–50%, *p* < 0.05) in A2058 cells treated with HILIC fraction D.

## 3. Discussion

Marine sponges, together with their symbiotic communities, represent a rich source of bioactive compounds displaying a wide range of potential applications in the biotechnological field [40,41,42,43]. Here, we evaluated the cytotoxicity and anti-proliferative capabilities of the crude extract and enriched fractions obtained from *H.* (*Halichoclona*) *vansoesti*, an alien sponge collected from the Strait of Messina (Faro Lake, Messina, Italy) [44]. This sponge species, already collected from Caribbean waters [45,46], was identified for the first time in the Mediterranean Sea by Bertolino et al. [33]. So far, several alien species have been recorded in the Strait of Messina, including macrophytae, polychaetes, molluscs, and sponges [44,46,47]. 

As reported in Bertolino et al. [33] the mechanism by which this species was introduced into the Mediterranean Sea from the Caribbean and Brazilian coasts is not clear. One of the factors could be due to intense maritime traffic, as well as the increasing number of bivalve farms often associated with organisms coming from the Atlantic Ocean. The presence of this sponge species in this lake could also be linked to global changes, such as climate change, which, together with changes from a socioeconomic point of view, are considered the main drivers for the introduction of alien species and their biodiversity in the future. 

The use of alien and invasive species for experimental purposes might be a concrete and sustainable research line to avoid the excessive harvesting of Mediterranean native species. 

Applying a bioassay-guided fractionation approach, we highlight possible anti-cancer and pharmacological properties of *H.* (*Halichoclona*) *vansoesti* metabolites against human melanoma cells. MTT assay showed that the enriched SPE fraction eluted with acetonitrile (CH_3_CN 100%, fraction HRX-D) was the most interesting, since low cytotoxicity was measured on the normal cell line (PNT2), while a dose-dependent effect on cell viability was recorded on the A2058 cancer cell line. Interestingly, when the fraction of interest (D) was further fractionated, MTT indicated that the cytotoxic effect in melanoma cells was spread between two new fractions (C and D). A chemical analysis of these two active samples clearly indicated the presence of different classes of sphingolipids. Fraction C was composed of the more complex lipid, cerebrosides, whereas fraction D contained sphingolipids of the halisphingosine family.

A sponge belonging to the *Haliclona* genus, *Haliclona* (*Reniera*) *tubifera* (George & Wilson, 1919), from the Brazilian coastline, was a source of two modified C18 sphingoid bases, namely (2R,3R,6R,7Z)-2-aminooctadec-7-ene-1,3,6-triol and (2R,3R,6R)-2-aminooctadec-1,3,6-triol (29,39). An ethyl acetate fraction of this sponge, containing sphingosine-derived content, showed cytotoxic effects on glioma (U87) and neuroblastoma (SH-SY5Y) cells, showing an IC_50_ < 15 μg/mL on both human cancer cell lines, as well as anticoagulant properties, increasing the recalcification time of human blood [22]. Safingol is a saturated analogue of sphingosine of the family of sphingolipids, whose cytotoxic effect consisted of inhibiting sphingosine kinase. In that way it was possible to prevent the formation of sphingosine-1-phosphate, which has a key role in in cell proliferation and angiogenesis, as well as cell death through protein kinase C inhibition [48,49].

Several sphingoid-based compounds were tested against seven human cancer cell lines, such as MCF-7 (mammary gland adenocarcinoma), A-549 (non-small cell lung cancer), MDA-MB-231 (mammary gland adenocarcinoma), HeLa (cervical adenocarcinoma), HTC-116 (human colon carcinoma), Jurkat (acute T-lymphoblastic leukemia), Caco-2 (human colon carcinoma), and the non-cancerous cell line NiH 3T3 (mouse fibroblasts), showing antiproliferative and cytotoxic activities [50]. Aiming to deeply investigate the gene pathway inducing cell death, a PCR array was performed on A2058 cells treated with active fractions C and D at IC_50_ concentrations, containing cerebrosides and halisphingosines, respectively. The PCR array showed that fraction D, enriched with sphingosine derivatives, activated a well-defined molecular response. In fact, we found that cells primarily undergo an autophagic cascade by activating several genes, such as ATG16L1, ATG5, and BECN1. These latter genes are involved in the nucleation and expansion of the autophagosome that subsequently merges with the lysosomes to initiate the degradation of dysfunctional organelles or microbes. Consequently, cells start a “self-eating” process that is normally used to counteract energy loss occurring during metabolic stress and disease (e.g., cancer, neurodegeneration, inflammation, and aging) [51,52,53,54]. 

An increase in the gene expression of *Fas* and its ligand (*FasL*) was also observed after 1 h of treatment at 1 µg mL^−1^ with the active fraction D. Fas, a type I transmembrane protein and member of the TNF receptor family, mediates the extrinsic apoptosis since it normally induces programmed cell death (PCD) through the binding of the cytokine FasL. This interaction triggered several cytoplasmic signal transducers recruiting pro-caspase 8 that, in turn, converts into its active form [55]. Contemporarily, a strong upregulation of *BCL2L11* (BIM), *APAF1*, and *CASP9* was observed. BCL2L11 is a pro-apoptotic protein that allows the permeabilization of the mitochondrial outer membrane and the release of several soluble proteins (e. g. cytochrome c) [56]. This change in the mitochondrial status induced the activation of APAF1 and oligomerization to CASP9 (apoptosome), which is the initiator caspase leading to the activation of the apoptosis executioner (CASP3) [56,57,58]. Real-time qPCR data indicated that the fraction under analysis might induce PCD in human melanoma cells (A2058), through a crosstalk between the extrinsic pathway, mediated by TNF receptors and the catalytic activity of caspase 8, and the mitochondria, via activating pro-apoptotic BCL-2 proteins and CASP9. These data were well confirmed by cell cycle analysis by flow cytometry. 

A similar apoptotic response has been recently observed in primary neurons treated for 24 h with cadmium at a concentration range of 5–20 µM [59]. Interestingly, CASP3 was not found to be particularly upregulated, since the fold regulation value was below the established cut-off. This result suggested that cells, in response to the harmful signals induced by the active fraction (D), could undergo apoptosis without completing the cell death process, probably due to the short incubation time (1 h). In fact, certain survival cues were also activated, with a gene expression increase in several defensive genes, such as *BCL2L1*, *EIF5B*, *XIAP*, *BIRC2*, *IGF1*, and *NFKB1* [56]. 

Corroborating the hypothesis of a pro-apoptotic and immunogenic cascade induced by sample D in human melanoma cell lines was the fact that several proteins promoting angiogenesis, such as angiopoietin-1 and angiopoietin-2, which inhibit vascular inflammation and prevent endothelial death [60], were significantly reduced. Moreover, G-CSF and GM-CSF, two linked protein factors that normally promote angiogenesis and tumor development, were also targeted [61], as was chemokine I-309, the binding of which to endothelial cells induces chemotaxis, invasion, and differentiation [62]. ImmunoArray analysis also indicated a decrease in the protein levels of MMP-9 and VEGF R3, both linked to the formation of blood vessels and cancer metastasis [63,64]. Interestingly, we corroborated the hypothesis raised by the gene expression analysis, suggesting that cells could undergo a PCD cascade without completing it, since some survival proteins, such as angiostatin, endostatin, and the cytokine IL-10 [65,66], were also found to be significantly reduced in treated A2058 cells.

Our data showed a significant inhibition of the expression of pro-angiogenic factors mediated by fraction D. In cancer, high levels of tissue inhibitors of metalloproteinases (TIMPs) are notable because they downregulate the activity of MMPs. For this reason, the angiogenic response can be strictly related to the modulation of genes associated to invasion and the balance between pro- and anti-angiogenic factors. Linked to this, the downregulation of pro-inflammatory chemokines and cytokines was evaluated from the molecular point of view. During tumor progression, these molecules are directly responsible for angiogenesis.

Overall, the present study explored the cerebrosides and sphingolipids derived from the sponge *H.* (*Halichoclona*) *vansoesti* for their potential chemopreventive and angiopreventive action, showing that they inhibited proliferation and promoted immunogenic cell death. 

The Caribbean region is a hotspot for biodiversity, and several examples of bioactive compounds have been reported in the literature, not only from sponges but also algae, corals, mollusks, microorganisms, cyanobacteria, and dinoflagellates [67]. The lipophilic extract of the Caribbean sponge *Cribrochalina vasculum* showed the presence of four new bioactive acetylene metabolites, namely (3R)-hydroxy-14-methyldocos-(4E)-en-1-yne, (3R)-hydroxy-16-methyleicos-1-yne, (3R)-hydroxy-19-methyleicos-1-yne, and docosa-(3E, 15Z)-dien-1-yne [68]. Bioactive metabolites were isolated from the Caribbean sponge *Aka coralliphagum*, such as the sulfated compounds siphonodictyals, corallidictyals C and D, and siphonodictyal G, with antimicrobial activity against bacteria, yeasts, and fungi, as well as antiproliferative activity on cultures of mouse fibroblasts [69]. Rodríguez-Berríos et al. [67] reviewed research from 1981 to 2020, reporting about ninety compounds (mainly polyketides) isolated in the Caribbean region, of which eighty-two showed biological activity. These results prompted the researchers to explore this peculiar marine environment to search for new natural bioactive compounds. Thanks to its high chemical biodiversity, the marine environment can be considered an amazing source of discoveries of bioactive compounds as potential drugs. Our in vitro study underlines the potential of the *H. vansoesti* metabolites as a resource of compounds for biotechnological applications in the medical field. Indeed, we demonstrated that the compounds reported in this study, namely cerebrosides and sphingolipids, were anti-proliferative against melanoma, suggesting their possible use as inspiration molecules in the design of new anticancer agents, not only as a monotherapy but also in combination with established treatment modalities, to enhance their action.

## 4. Materials and Methods

### 4.1. Chemical Extraction and Fractionation

*Haliclona* (*Halichoclona*) *vansoesti* [44] was collected from the Strait of Messina (Faro Lake, Messina, Italy) and stored at −80 °C, until use [33]. The sponge was previously identified, starting from four samples, by morphological observations of its body, shape, and skeleton, and using a 28S rRNA molecular marker [33]. Prior to chemical extraction, wet samples (~80 g) were lyophilized under a freeze dryer. Methanol (Merk Life Science S.r.l., Milan, Italy) extraction was performed on dried sponge tissues (~7 g) using a Precellys Evolution tissue homogenizer equipped with a Cryolys Evolution cooling system (Bertin Italia, Genoa, Italy) to obtain 1.2 g of crude extract. The protocol of extraction was already set up for marine samples [3,20] and consisted of a run at 6200 rpm (3 cycles × 30 s) at a temperature of 16 °C to prevent degradation, followed by centrifugation of the sample at 3450 rpm for 10 min at 4 °C. The extract was filtered and dried in a rotatory evaporator at room temperature. About 100 mg of raw extract was subjected to SPE on a GX-271 ASPEC Gilson apparatus (Gilson Italy, Cinisello, Italy) by using CHROMABOND^®^ HRX cartridges (6 mL/500 mg, Macherey-Nagel, Düren, Germany) [3]. Briefly, this fractionation yielded five samples (A: 44.2 mg, B: 14.8 mg, C: 10.6 mg, D: 2.1 mg and E: 10.9 mg) eluted with H_2_O, CH_3_OH/H_2_O 1:1, CH_3_CN/H_2_O 7:3, CH_3_CN, and CH_2_Cl_2_/CH_3_OH 9:1, respectively. Total extract and the enriched fractions B–E were tested. After preliminary ^1^H NMR of the SPE-HRX fraction D (1.9 mg), the sample was subjected to a further solid-phase extraction using hydrophilic interaction chromatography (or hydrophilic interaction liquid chromatography, HILIC), using a prepacked column with CHROMABOND^®^ HILIC cartridges (6 mL/500 mg, Macherey-Nagel, Düren, Germany) and the automated GX-271 ASPEC Gilson system [20]. In detail, the cartridge was conditioned with 2 mL of milli-q water and equilibrated with 10 mL of tetrahydrofuran (THF)/n-hexan 50:50 (*v*/*v*). The sample was suspended in 1 mL of THF/n-hexan 50:50 (*v*/*v*) and sonicated for a few seconds in an ultrasonic bath before being loaded onto the column. Elution steps led us to obtain five new fractions (A–E) each eluted with 6 mL of THF/n-hexane 50:50 *v*/*v* (A: 0.8 mg), THF 100% (B: 0.2 mg), THF/MeOH 90:10 *v*/*v* (C: 0.1 mg), THF/MeOH 80:20 *v*/*v* (D: 0.2 mg), and THF/MeOH 10:90 *v*/*v* (E: 0.1 mg), respectively. All HILIC fractions were tested and the active samples C (0.1 mg) and D (0.2 mg) were analyzed by mono bidimensional NMR on a Bruker DRX 600 MHz spectrometer (Bruker Bio-Spin, Fällanden, Swiss) equipped with a TXI CryoProbe in CD_3_OD (δ values of 3.34 and 49.0 ppm) and a HRESI-MS/LCMS-MSMS on a Q-Exactive™ Hybrid Quadrupole-Orbitrap™ Mass Spectrometer (Thermo Scientific, Waltham, MA, USA) coupled with a 1290 Infinity UPLC System (Agilent Technologies, Santa Clara, CA, USA) (Figures S8–S10, S14 and S15) with the ESI source in the negative mode using an RP-column (Phenomenex Luna C18 150 × 5 mm, 5 µm), with water and MeOH as solvent A and B, respectively, starting in an isocratic condition with 10:90 A/B (*v*/*v*) for 15 min followed by a gradient up to 100% of B in 10 min, with a flow rate of 0.8 mL/min. 

MS raw data are available and accessible at the following link: https://cloud.icb.cnr.it/s/Y7rS39KZZc9A9A8 (accessed on 1 March 2024). 

In order to obtain a more accurate characterization of cerebrosides, approximately 200 mg of raw extract was submitted to the SPE fractionations (HRX and HILIC) to yield 0.5 mg of faction C. This sample was further purified by a silica gel small column using an elution gradient from 7 to 15% of methanol in dichloromethane to produce subfraction 1 (**1b**, **c**, and **e**) and 2 (**1a** and **d**). Chromatographic fractionation was monitored by TLC eluted with DCM/MeOH 85:15 (see Figure S23)

### 4.2. Cell Maintenance and Treatments

The following cell lines were used: PNT2 (normal prostate epithelium immortalized with SV40) grown in RPMI 1640; A2058 (human melanoma epithelial cell line) grown in Dulbecco’s modified Eagle’s medium (DMEM). Media were enriched with 10% (*v*/*v*) of fetal bovine serum (FBS), 2 mM of L-glutamine, 100 units mL^−1^ of penicillin and streptomycin. Once cultures reached confluence (approximatively every 3 days), cells were detached by using trypsin, and the culture medium was changed. Before the experiments, cells were placed in 96-well plates and kept overnight in a thermostatic chamber in a 5% CO_2_ atmosphere at 37 °C for the attachment. For viability assays, the total extracts and fractions were suspended in dimethyl sulfoxide (DMSO) at a final concentration of 1% (*v*/*v*). Three standard concentrations were used for the analysis: 1, 10, and 100 µg mL^−1^. The concentrations in the case of the subfractions were 0.1, 1, and 10 µg mL^−1^, respectively. 

### 4.3. Cytotoxicity Assay 

The crude extract and fractions B, C, D, and E were tested on human cell lines at three different concentrations: 1, 10, and 100 µg/mL^−1^. No study was performed to verify that the biological activity came from the sponge and not from some endophytic microorganisms, such as fungus or bacteria. The cytotoxic effect of sponge extracts and fractions was evaluated by 3-(4,5-Dimethylthiazol-2-yl)-2,5-Diphenyltetrazolium Bromide (MTT) assay (Applichem A2231, Darmstadt, Germany). After 48 h of treatment, cells were treated with 10 μL of MTT (5 mg mL^−1^) and incubated in the dark for 3 h at 37 °C. Then, 100 µL of isopropanol was added to the cells and incubated under agitation for 1 h to let the purple formazan crystals dissolve. The absorbance was detected on a microplate reader at a wavelength of 570 nm (TECAN, Life Sciences). The anti-proliferative activity was measured as percentage of cell viability considering the ratio of the absorbance of each sample (reported as a mean), as well as for the absorbance of controls.

### 4.4. RNA Extraction, cDNA Synthesis, and RT^2^ Profiler PCR-Array

A2058 cells were pipetted in 6-well multiwells and grown overnight in 2 mL of culture media to extract RNA. Cells were treated for two hours with the fraction of interest at the IC_50_ concentration. Treatments and controls (grown in medium without the addition of the fractions) were carried out in triplicates. After two hours of exposure time, cells were washed using cold phosphate-buffered saline (PBS) and lysed in 500 µL of TRIsure reagent (Bioline, London, UK) for each mL of culture medium. Then, the RNA extraction was conducted, following the manufacturer’s instructions. RNA quantity (measured as ng µL^−1^) and purity (calculating the ratios A260/A280, A260/A230) was assessed through a NanoDrop spectrophotometer (Thermo Scientific). About 200 ng of RNA were retrotranscribed by using the RT^2^ first strand kit (Qiagen, Venlo, The Netherlands). RT-qPCR experiments were performed in triplicates by using a RT^2^ profiler PCR-array kit for cell death pathway identification (Qiagen), run on a ViiA7 (Applied Biosystems, Waltham, MA, USA) through a standard fast PCR cycling protocol, including a denaturation step at 95 °C for 10 min, 40 amplification cycles at 95 °C for 15 s and 60 °C for 1 min, and a final extension step at 72 °C for 10 min, by using 10 μL of the solution for the final reaction. Amplification data were collected by using the ViiA™ 7 software v1.0. (accessed 1 February 2024; Applied Biosystems). The cycle threshold (C_t_) values were then analyzed through PCR array data analysis using online software (https://dataanalysis2.qiagen.com/pcr, Qiagen; accessed on 10 January 2024). Fold-change values (gene expression ratios) were calculated on the basis of the 2^(−∆∆Ct)^ method [70]. The reference genes [71] used for data normalization were *actin-beta* (ACTB), *beta-2-microglobulin* (B2M), *glyceraldehyde-3-phosphate dehydrogenase* (GAPDH), *hypoxanthine phosphoribosyltransferase 1* (HPRT1), and *ribosomal protein, large subunit P0* (RPLP0), and their expressions were found to be unchanged in control and treated cells. To show a more readable gene expression data, fold changes (x) were converted to fold regulation values as (−1/x). The cut-off was set at ±2.

### 4.5. Protein Extraction and ImmunoArray

A2058 cells were seeded in 6-well multiwells and left overnight in a thermostatic chamber at 37 °C for the attachment process. Cells were treated with the fraction of interest at the IC_50_ concentration for 24 h at 37 °C. Treatments and controls (medium without the compound) were performed in triplicates. Protein extraction was performed by adding 1 mL of 1× lysis buffer (Raybiotech, Norcross, GA, USA). After washing A2058 cells with cold PBS, cell debris and supernatants were divided through centrifugation at 12,000× *g* for 5 min at 4 °C and stored at −20 °C until use. Then, ImmunoArray experiments were applied on 400 µL of lysates using a RayBio^®^ C-Series Human Angiogenesis Antibody Array kit, according to the manufacturer’s instructions. For protein detection, membranes were incubated with 1 mL of biotinylated antibody cocktail and 2 mL of 1× HRP-streptavidin. Array membranes were analyzed under a ChemiDoc imaging system (Bio-Rad) and signal intensity for each antigen-specific antibody spot was converted into numerical values through a 2D densitometry approach. Ratios between treatment and controls were applied to measure the relative expression of each protein.

### 4.6. Statistical Analyses

Multiple Student’s *t* tests were applied to evaluate the differences between the groups (mean ± SD) of biological replicates (N = 3). Statistical analyses and dose–response curve were designed using GraphPad Prism v9.0 for Windows, GraphPad Software, La Jolla, CA, USA, www.graphpad.com. For RT-qPCR, Student’s *t* tests of the triplicate 2^–∆Ct^ values for the genes under analysis in each control and treated groups comparison were performed using the available online software used for data analysis (https://dataanalysis2.qiagen.com/pcr, Qiagen).

## Figures and Tables

**Table 1:** Rt and main *m*/*z* ion peak and related fragments.

Rt (min)	*m*/*z* Ion Peak [M-H]^−^/[M+Cl]^−^	*m*/*z* Fragment [M-H-Glc]^−^	Molecular Formula	Exact Mass
19.20	732.56/768.54	570.52	C_40_H_79_NO_10_	733.5704
20.97	712.54/748.53	550.49	C_40_H_75_NO_9_	713.5442
22.49	726.56/762.54	564.50	C_41_H_77_NO_9_	727.5962
23.02	760.60/796.59	598.54	C_42_H_83_NO_10_	761.6017
23.79	740.57/776.56	578.52	C_42_H_79_NO_9_	741.5755
26.64	816.66/852.65	654.61	C_46_H_91_NO_10_	817.6643
27.29	830.67/866.65	668.62	C_46_H_89_NO_11_	831.6436

**Table 2:** Relevant NMR Data ^a^ of compounds belonging to HILIC fraction C in CD_3_OD at 600 MHz.

	1a, 1d		1b, 1c, 1e	
Position	δ_H_ (m, *J* in Hz)	δ_C_, Type	δ_H_ (m, *J* in Hz)	δ_C_, Type
1	4.08 (dd, 10.5, 6.0) 3.83 (dd, 10.5, 3.8)	69.7, CH_2_	4.14 (overlap.) 3.75 (dd, 10.5, 3.8)	69.5, CH_2_
2	4.28 (m)	51.5, CH	4.02 (m)	54.4, CH
3	3.64 (t, 6.0)	75.2, CH	4.17 (overlap.)	72.4, CH
4	3.55 (m)	72.9, CH	5.51 (dd, 15.0, 7.0)	130.9, CH
5	1.44 (m)	32.6, CH	5.77 (dt, 15.0, 7.0)	134.3, CH
6			2.10 (m)	33.3, CH_2_
7			2.11 (m)	33.4, CH_2_
8, 9			5.45 (m)	131.0, CH
10			2.00 (m)	34.8, CH_2_
11			1.37 (m)	23.9, CH_2_
1′		176.5, C		176.5, C
2′	4.05 (dd, 8.0, 4.2)	72.7, CH	4.05 (dd, 8.0, 4.2)	72.7, CH
3′	1.64 (m)	35.2, CH_2_	1.64 (m)	35.2, CH_2_
4′	1.30 (m)	30.0, CH_2_	1.30 (m)	30.0, CH_2_
1″	4.31 (d, 7.7)	104.1, CH	4.31 (d, 7.7)	104.1, CH
2″	3.21 (m)	74.8, CH	3.21 (m)	74.8, CH
3″	3.38 (m)	77.5, CH	3.38 (m)	77.5, CH
4″	3.30 (m)	71.7, CH	3.30 (m)	71.7, CH
5″	3.31 (m)	77.3, CH	3.31 (m)	77.3, CH
6″	3.70 (dd, 12.0, 2.0) 3.90 (dd, 12.0, 3.0)	62.5, CH_2_	3.70 (dd, 12.0, 2.0) 3.90 (dd, 12.0, 3.0)	62.5, CH_2_

^a^ The structure assignment was obtained by ^1^H-^1^H COSY, TOCSY, HSQCedited, and HMBC correlations.

**Table 3:** Relevant NMR data ^a^ of compounds belonging to HILIC fraction D in CD_3_OD at 600 MHz.

Position	δ_H_ (m, *J* in Hz)	δ_C_, Type
1	3.74 (dd, 11.0, 3.7) 3.62 (m)	61.8, CH_2_
2	2.96 (m)	58.4, CH
3	3.66 (m)	69.7, CH
4	1.54–1.40 (m)	35.0, CH_2_
5	1.56–1.40 (m)	38.0, CH_2_
6	4.39 (m)	67.8, CH
7	5.35 (dd, 10.0, 9.0)	134.2, CH
8	5.44 (overlap.)	133.0, CH
9	2.08 (m)	28.6, CH_2_
10–17	1.40–1.25	
18	0.90 (t, 7.0)	14.0, CH_3_

^a^ The structure assignment was obtained by ^1^H-^1^H COSY and HSQC correlations.

## Data Availability

Data is contained within the article and Supplementary Materials. MS raw data are available and accessible at the following link: https://cloud.icb.cnr.it/s/Y7rS39KZZc9A9A8.

## Supplementary Materials

The following supporting information can be downloaded at: https://www.mdpi.com/article/10.3390/ijms25137418/s1.
