# Supplementary material for: Potential of Polar Lipids Isolated from the Marine Sponge Haliclona (Halichoclona) vansoesti against Melanoma
Source: Int J Mol Sci. 2024 Jul 6;25(13):7418. doi: 10.3390/ijms25137418 (PMC11242152; doi:10.3390/ijms25137418)
Supplement: Supplementary file 1 [file ijms-25-07418-s001.zip › ijms-3050776-supplementary.pdf]

Supplementary Material

Figure S1. ^{1}H NMR of fraction HRX-D (600 MHz, CD_3OD).

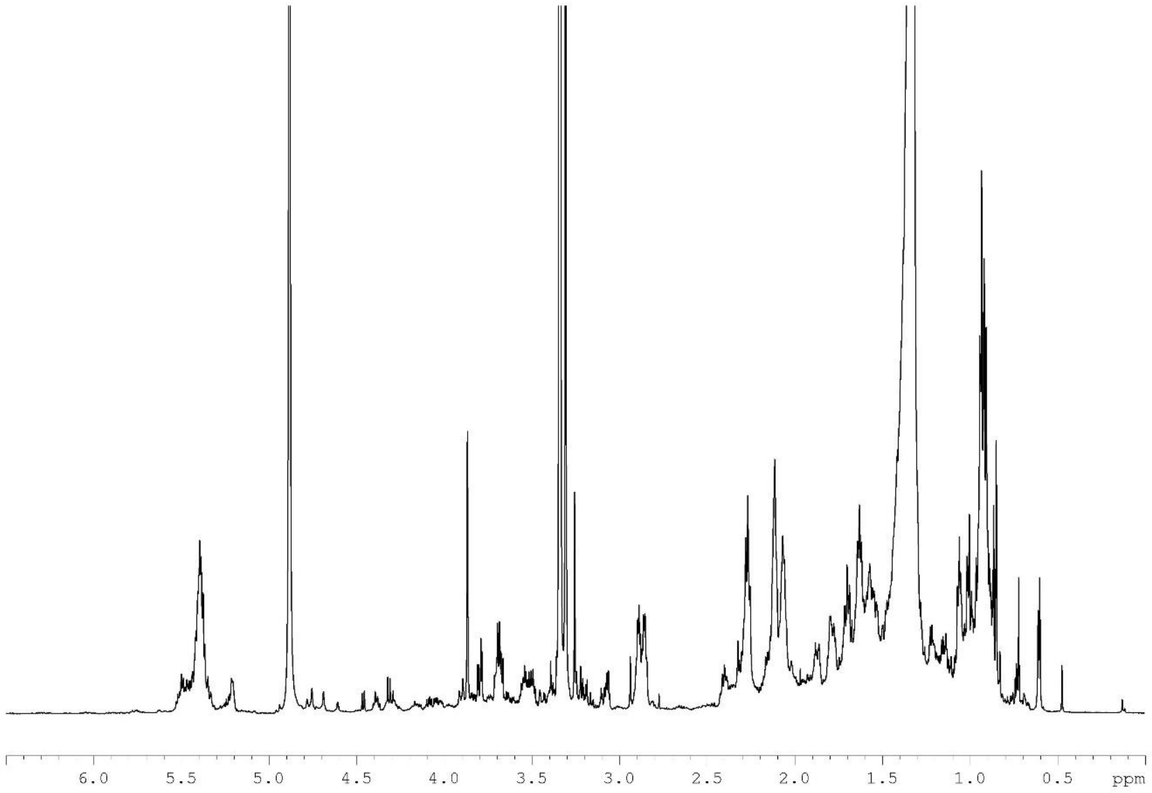

Figure S2 TLC analysis of HILIC-SPE samples A-E obtained from fractionation of fraction HRX-D, using $CHCl_3$/MeOH/water 65:25:4 as eluent.

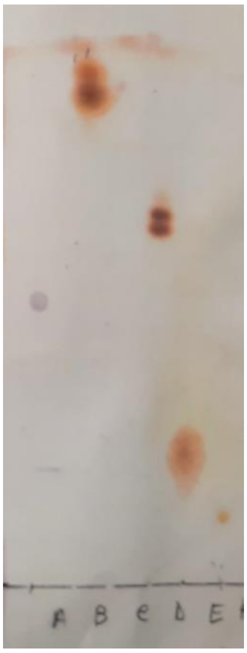

Figure S3. Fragmentation pattern of the main compounds belonging to HILIC fraction C.

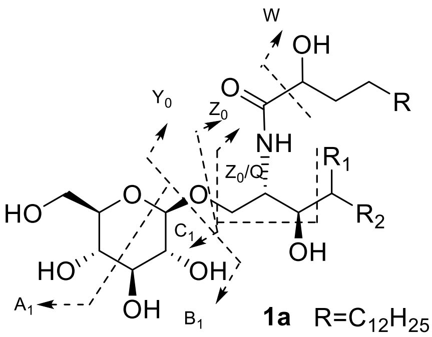

1a $R=C_{12}H_{25}$; $R_1=OH$ $R_2=C_{14}H_{29}$
1b $R=C_{12}H_{25}$; $R_1=H$; $R_2=C_{14}H_{25}$
1c $R=C_{13}H_{27}$; $R_1=H$; $R_2=C_{14}H_{25}$
1d $R=C_{14}H_{29}$; $R_1=OH$ $R_2=C_{14}H_{29}$
1e $R=C_{14}H_{29}$; $R_1=H$; $R_2=C_{14}H_{25}$

Figure S4. ^{1}H NMR of HILIC fraction C (600 MHz, CD_3OD).

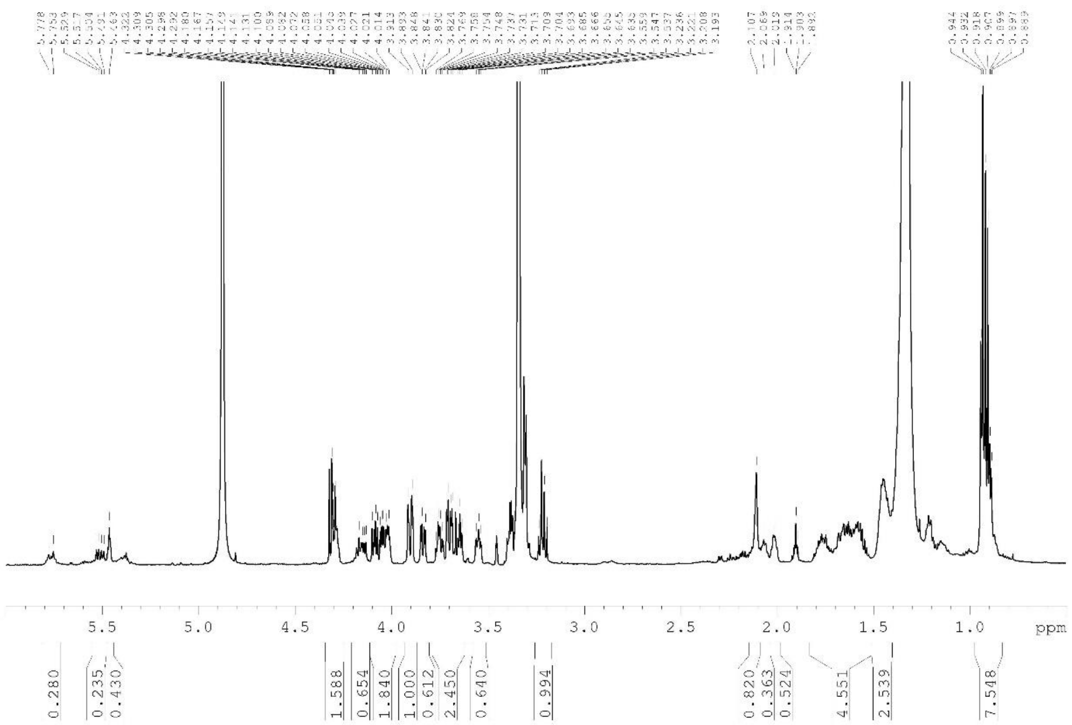

Figure S5. COSY NMR of HILIC fraction C (600 MHz, CD_3OD).

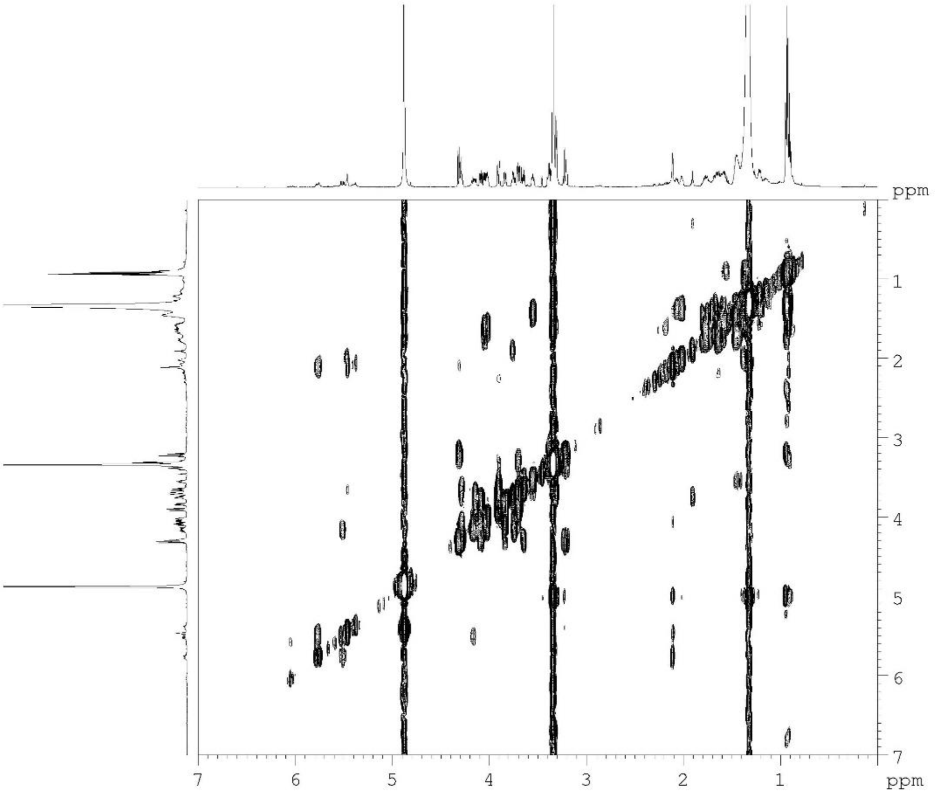

Figure S6. TOCSY NMR of HILIC fraction C (600 MHz, CD_3OD).

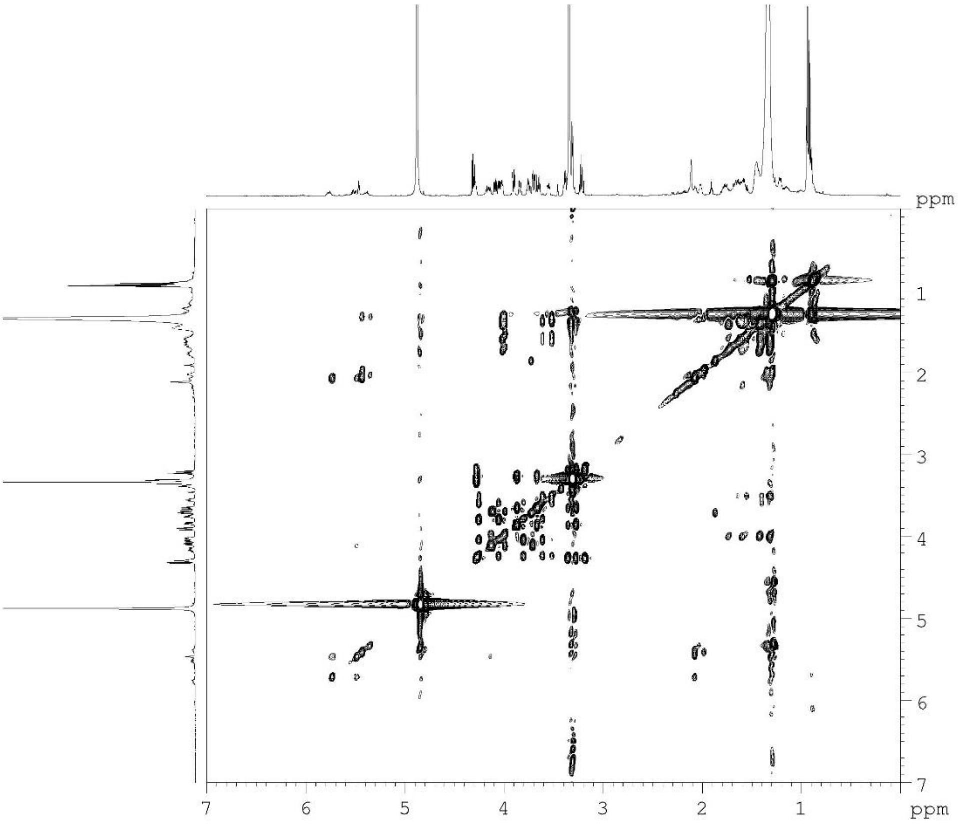

Figure S7. HSQCed NMR of HILIC fraction C (600 MHz, CD_3OD): CH_2 are shown in light gray, while CH and CH_3 signals appear in black.

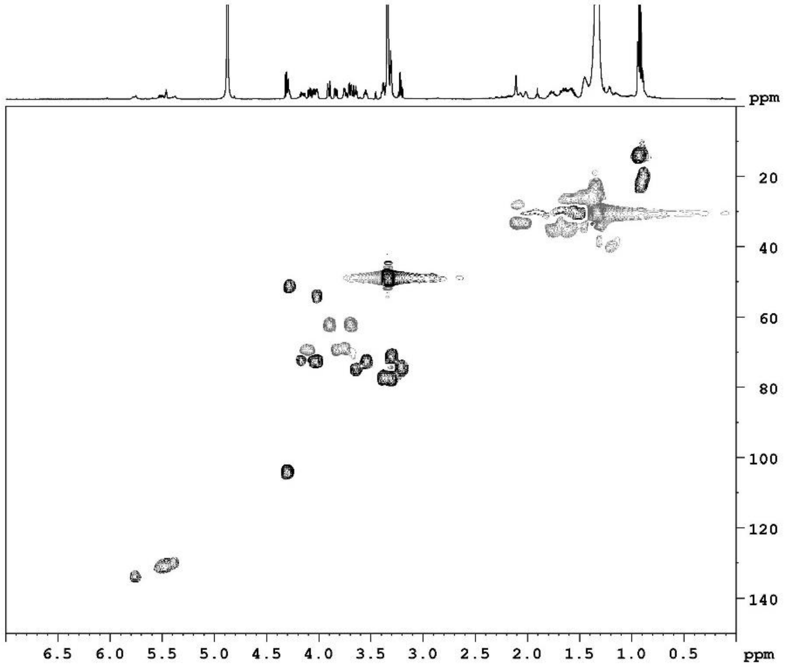

Figure S8. HMBC NMR of HILIC fraction C (600 MHz, CD_3OD).

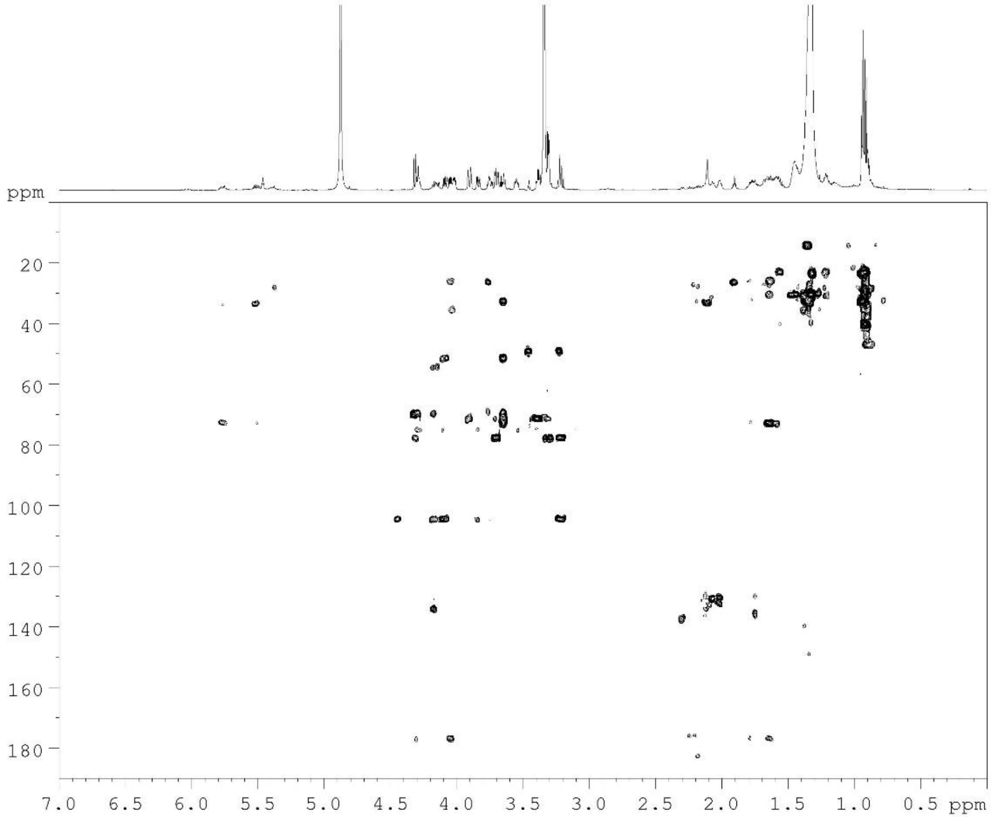

Figure S9. LC-MS-MSMS analysis of HILIC fraction C in HRESI negative mode.

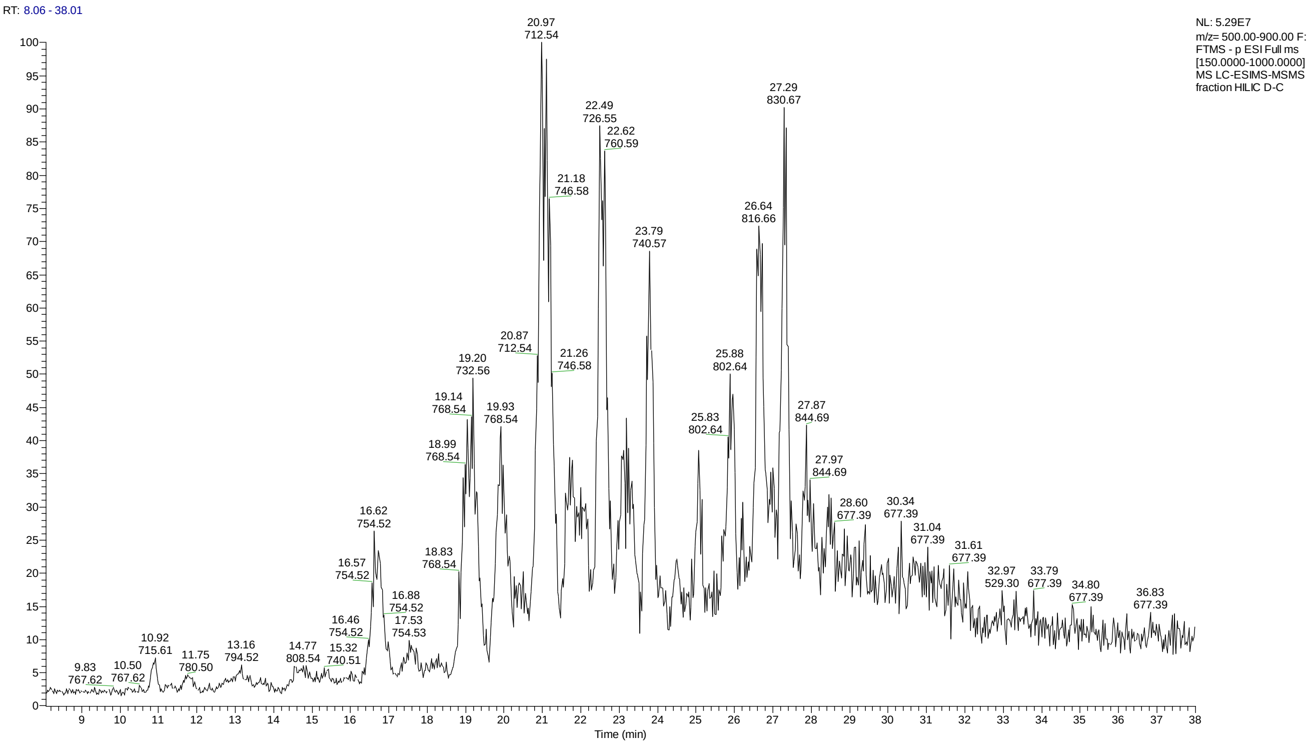

Figure S10. MSMS of the main compounds belonging to HILIC fraction C (diagnostic sugar fragment ion peak at *m/z* 179.06): A) MSMS spectrum of the ion peak at m/z 732.5628; B) MSMS spectrum of the ion peak at m/z 768.5428; C) MSMS spectrum of the ion peak at m/z 712.5388; D) MSMS spectrum of the ion peak at m/z 726.5551; E) MSMS spectrum of the ion peak at m/z 760.5957; F) MSMS spectrum of the ion peak at m/z 740.5691; G) MSMS spectrum of the ion peak at m/z 816.6561; H) MSMS spectrum of the ion peak at m/z 830.6730.

A)

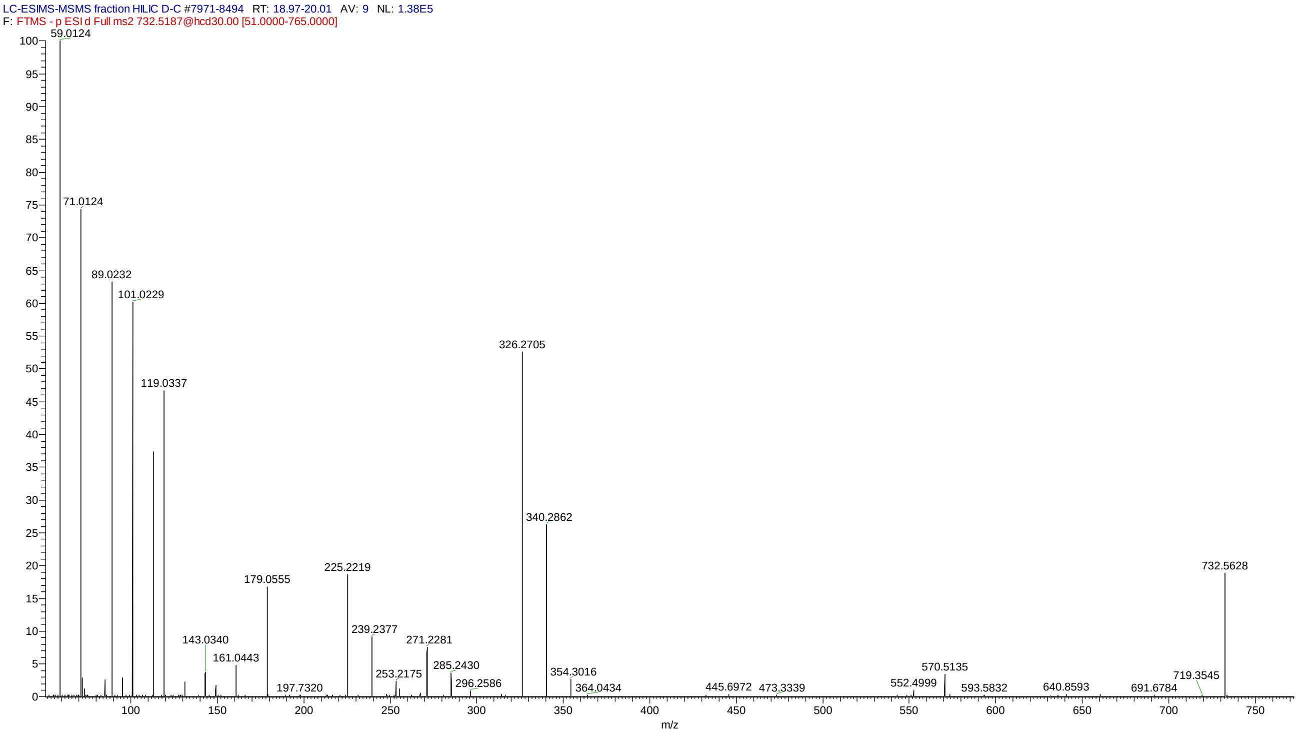

A1)

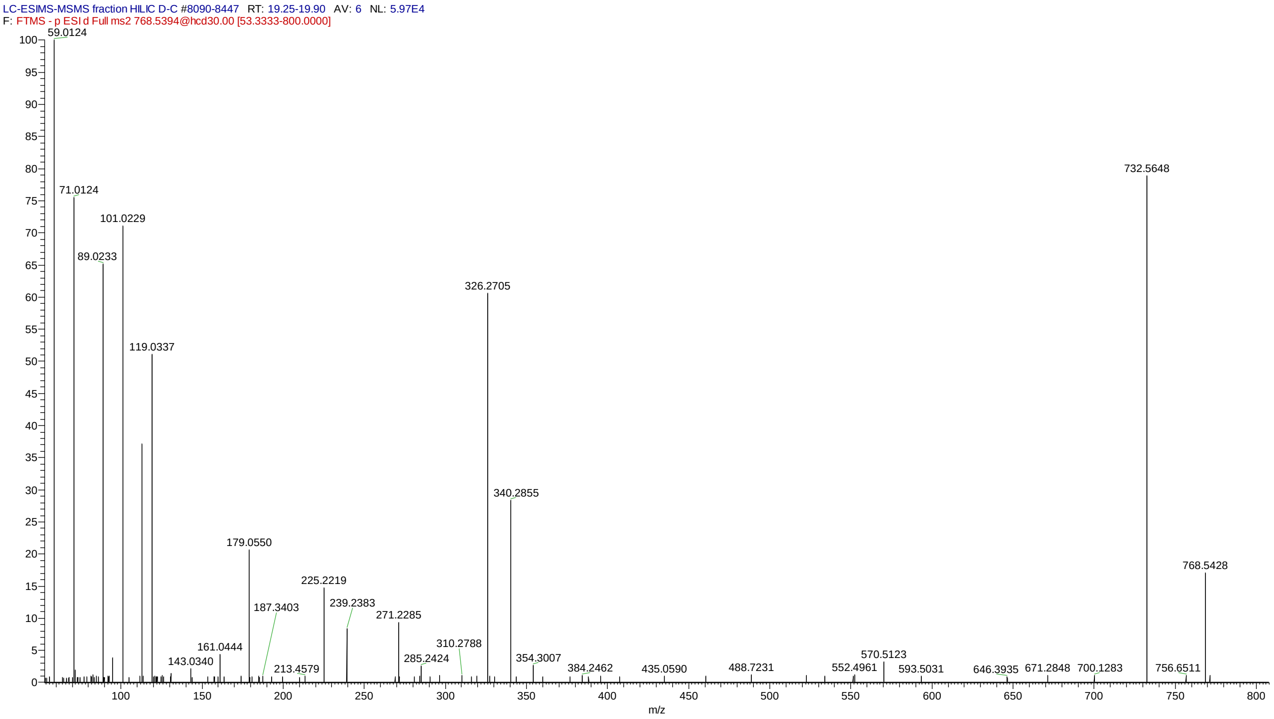

B)

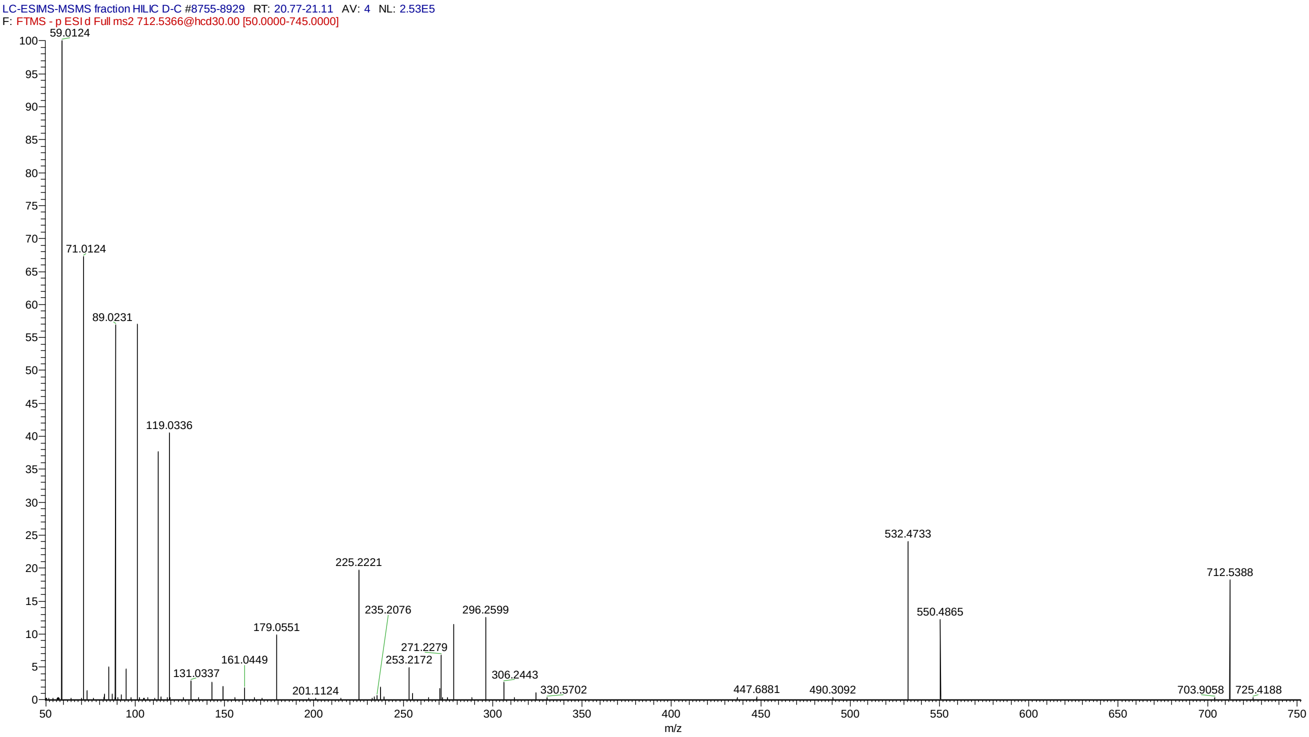
LC-ESIMS-MSMS fraction HILIC D-C #8755-8929 RT: 20.77-21.11 AV: 4 NL: 2.53E5
F: FTMS - p ESI d Full ms2 712.5366@hcd30.00 [50.0000-745.0000]
59.0124
71.0124
89.0231
119.0336
131.0337
161.0449
179.0551
201.1124
225.2221
235.2076
253.2172
271.2279
296.2599
306.2443
330.5702
447.6881
490.3092
532.4733
550.4865
703.9058
712.5388
725.4188
m/z

C)

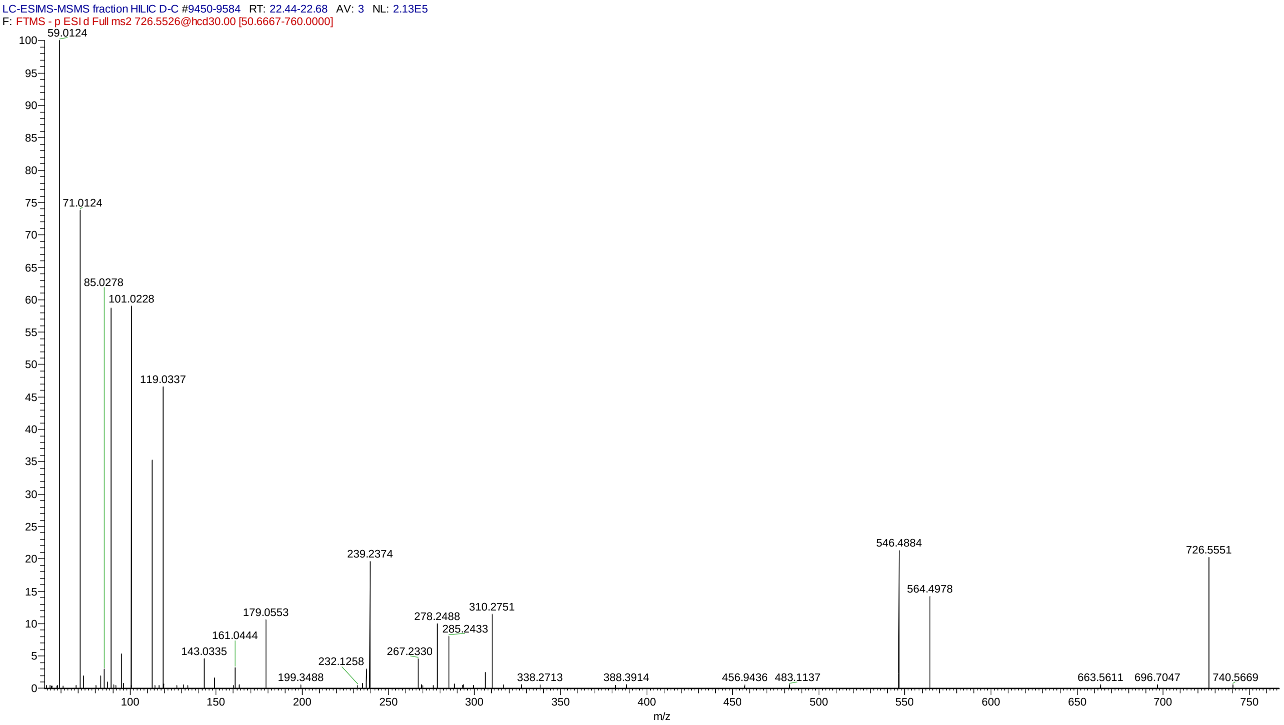
LC-ESIMS-MSMS fraction HILIC D-C #9450-9584 RT: 22.44-22.68 AV: 3 NL: 2.13E5
F: FTMS - p ESI d Full ms2 726.5526@hcd30.00 [50.6667-760.0000]
59.0124
71.0124
85.0278
101.0228
119.0337
143.0335
161.0444
179.0553
199.3488
232.1258
239.2374
267.2330
278.2488
285.2433
310.2751
338.2713
388.3914
456.9436
483.1137
546.4884
564.4978
663.5611
696.7047
726.5551
740.5669
m/z

D)

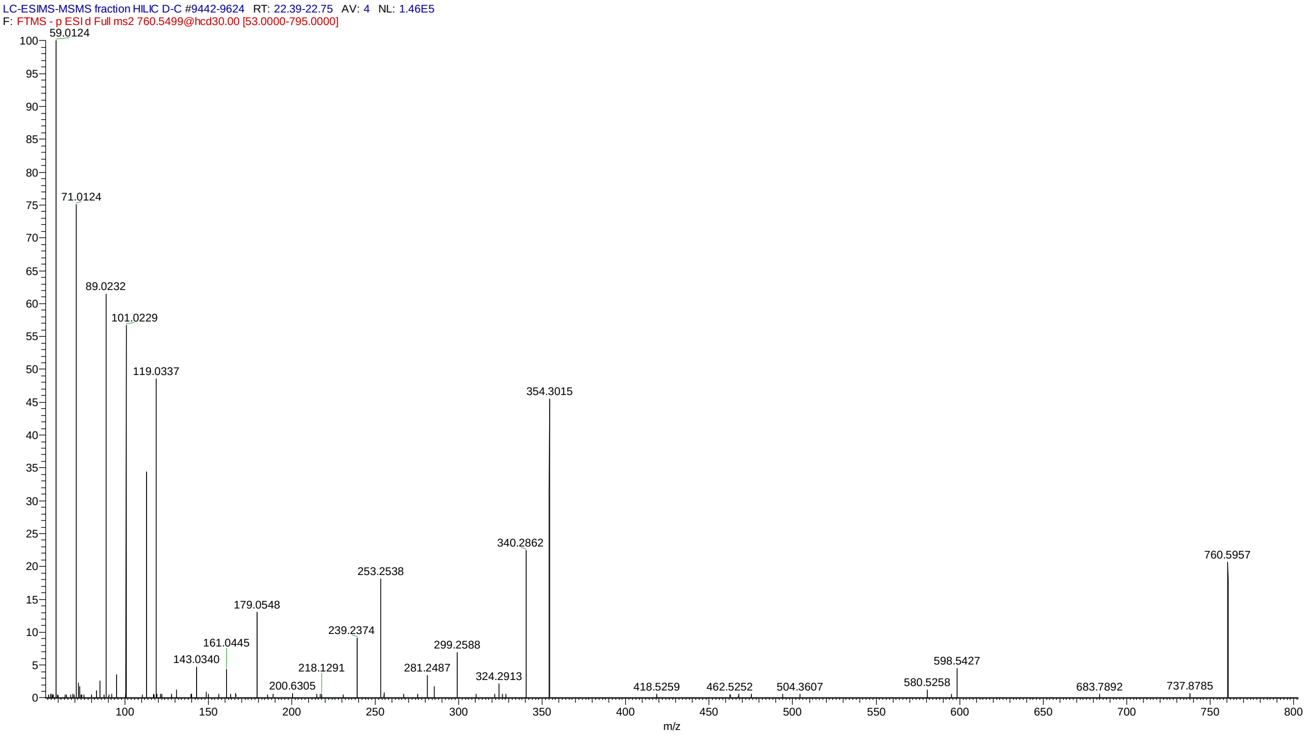
LC-ESIMS-MSMS fraction HILIC D-C #9442-9624 RT: 22.39-22.75 AV: 4 NL: 1.46E5
F: FTMS - p ESI d Full ms2 760.5499@hcd30.00 [53.0000-795.0000]
59.0124
71.0124
89.0232
101.0229
119.0337
354.3015
340.2862
760.5957
253.2538
179.0548
239.2374
161.0445
299.2588
143.0340
218.1291
281.2487
598.5427
324.2913
200.6305
418.5259
462.5252
504.3607
580.5258
683.7892
737.8785
m/z

E)

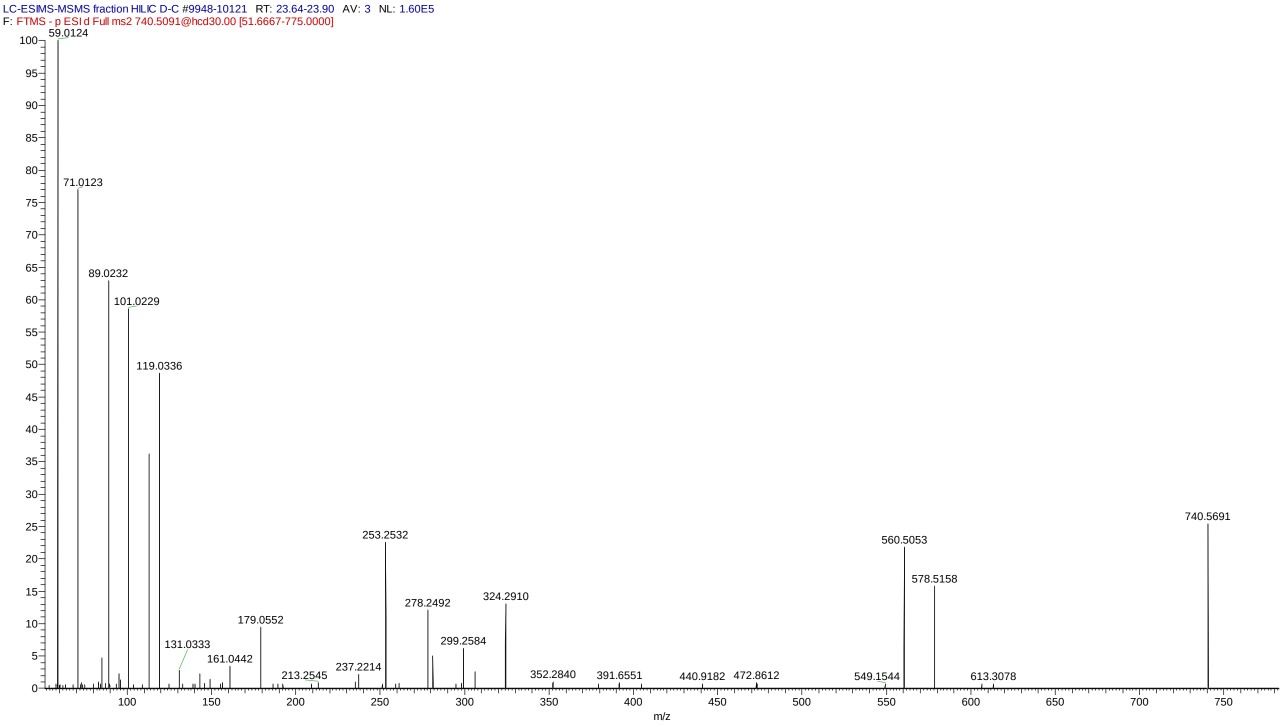
LC-ESIMS-MSMS fraction HILIC D-C #9948-10121 RT: 23.64-23.90 AV: 3 NL: 1.60E5
F: FTMS - p ESI d Full ms2 740.5091@hcd30.00 [51.6667-775.0000]
59.0124
71.0123
89.0232
101.0229
119.0336
740.5691
253.2532
560.5053
578.5158
278.2492
324.2910
179.0552
299.2584
131.0333
161.0442
237.2214
213.2545
352.2840
391.6551
440.9182
472.8612
549.1544
613.3078
m/z

F)

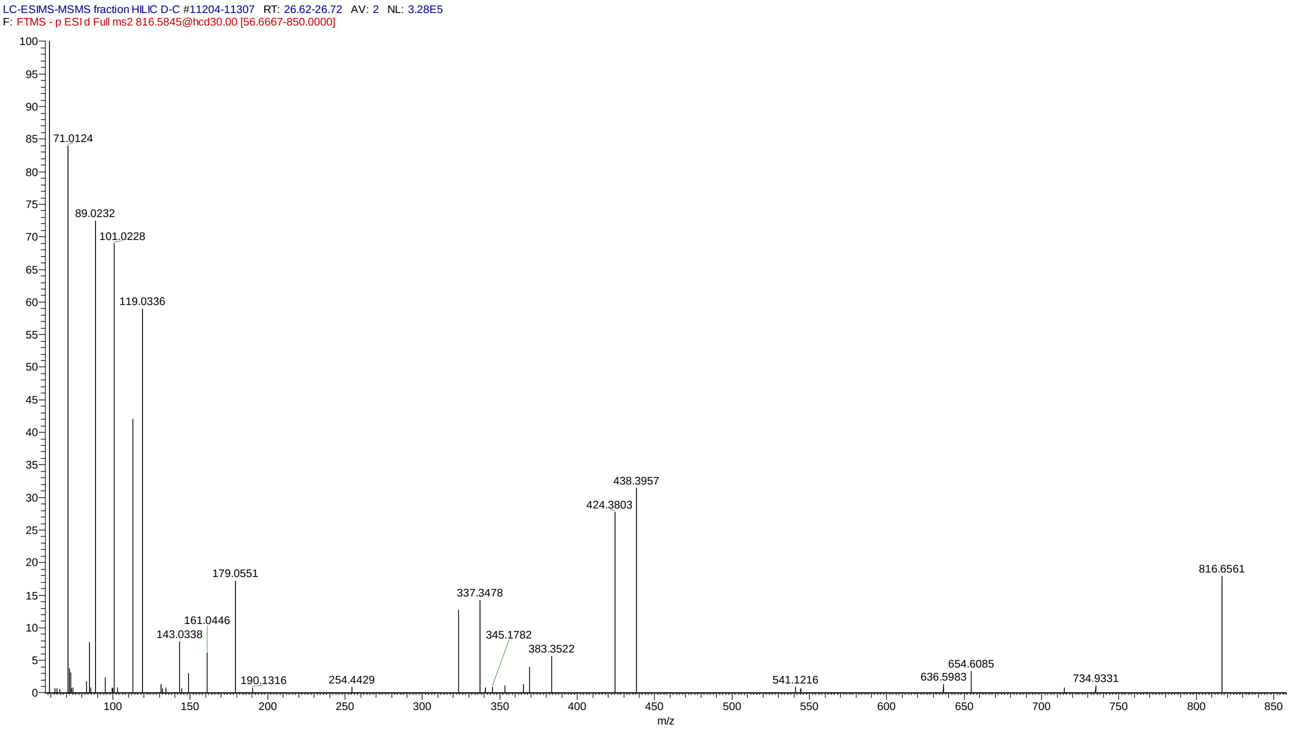
LC-ESIMS-MSMS fraction HILIC D-C #11204-11307 RT: 26.62-26.72 AV: 2 NL: 3.28E5
F: FTMS - p ESI d Full ms2 816.5845@hcd30.00 [56.6667-850.0000]
71.0124
89.0232
101.0228
119.0336
143.0338
161.0446
179.0551
190.1316
254.4429
337.3478
345.1782
383.3522
424.3803
438.3957
541.1216
636.5983
654.6085
734.9331
816.6561
m/z

G)

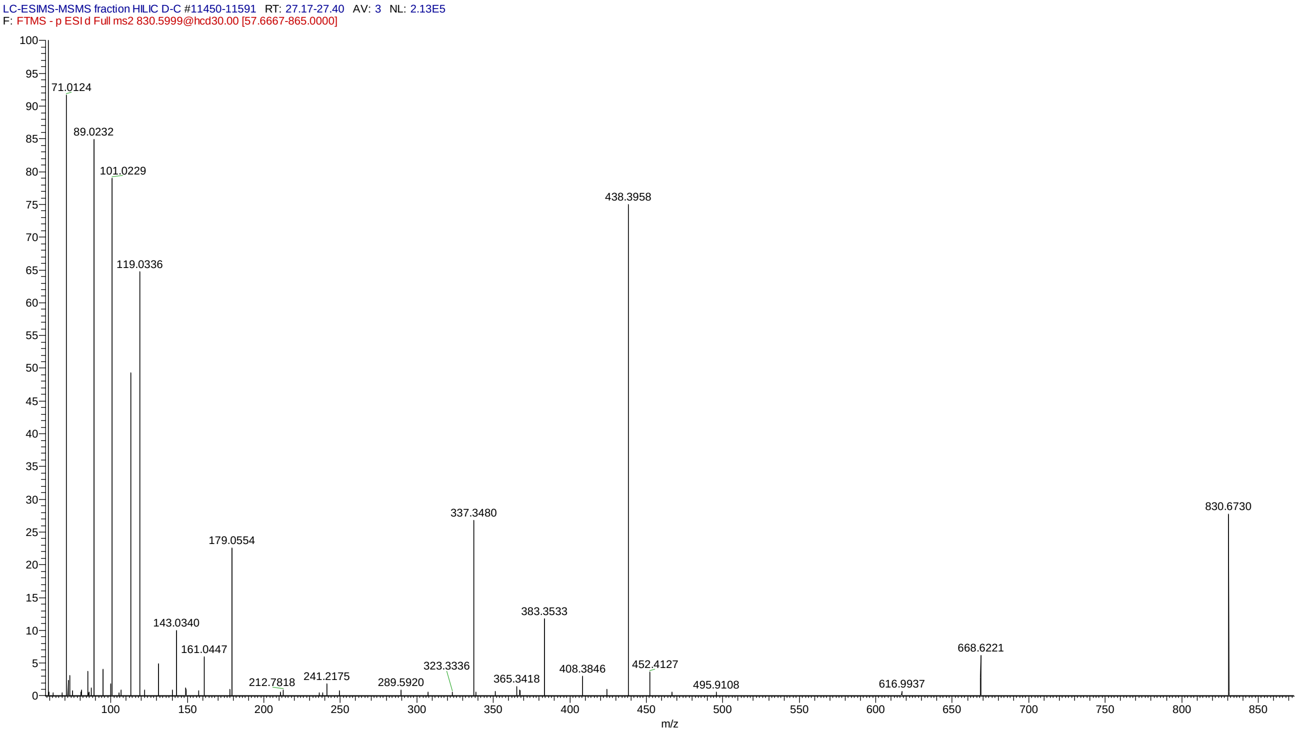
LC-ESIMS-MSMS fraction HILIC D-C #11450-11591 RT: 27.17-27.40 AV: 3 NL: 2.13E5
F: FTMS - p ESI d Full ms2 830.5999@hcd30.00 [57.6667-865.0000]
71.0124
89.0232
101.0229
119.0336
143.0340
161.0447
179.0554
212.7818
241.2175
289.5920
323.3336
337.3480
365.3418
383.3533
408.3846
438.3958
452.4127
495.9108
616.9937
668.6221
830.6730
m/z

Figure S11. ^1H NMR of HILIC fraction D (600 MHz, CD_3OD).

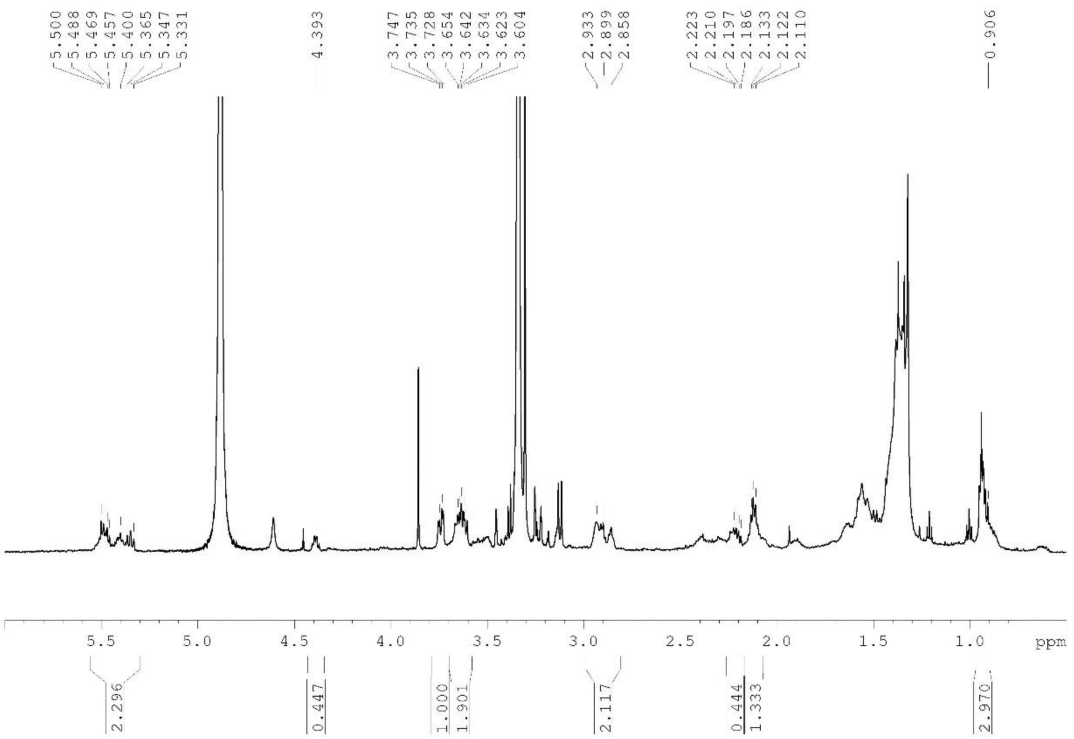

Figure S12. COSY NMR of HILIC fraction D (600 MHz, CD_3OD).

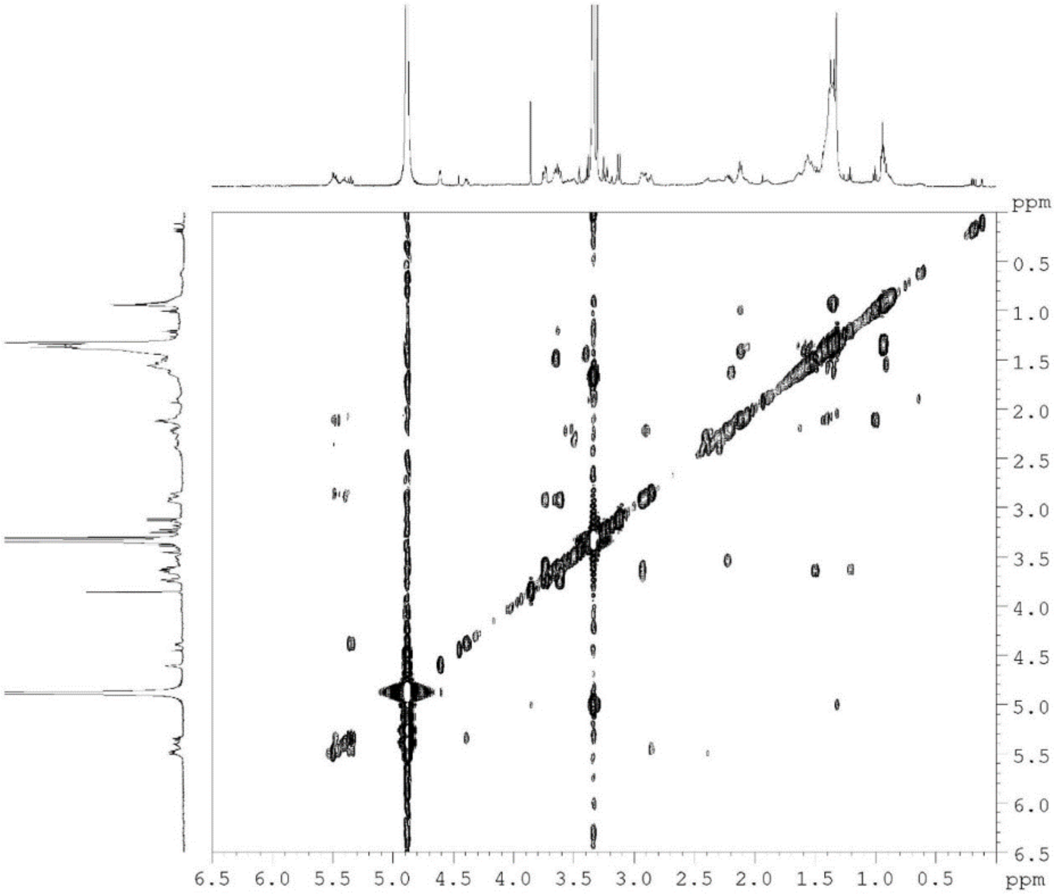

Figure S13. HSQCed NMR of HILIC fraction D (600 MHz, CD_3OD): CH_2 are shown in light gray, while CH and CH_3 signals appear in black.

.

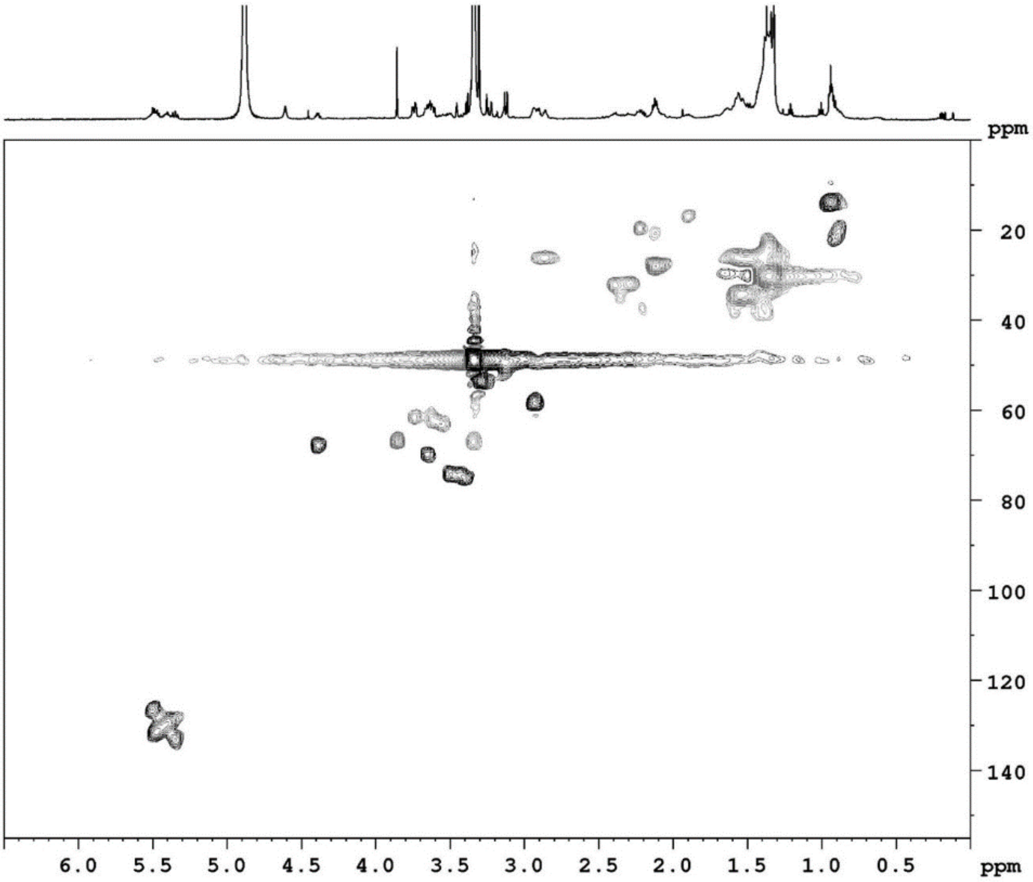

Figure S14. HR-ESI$^+$ MS of HILIC fraction D. Main *m/z* peaks in the region between 250 and 450. The ion peak relating to $[M+H]^+$, $[M-H_2O+H]^+$, $[M-2H_2O+H]^+$ of halisphingosine A are indicated in the spectrum.

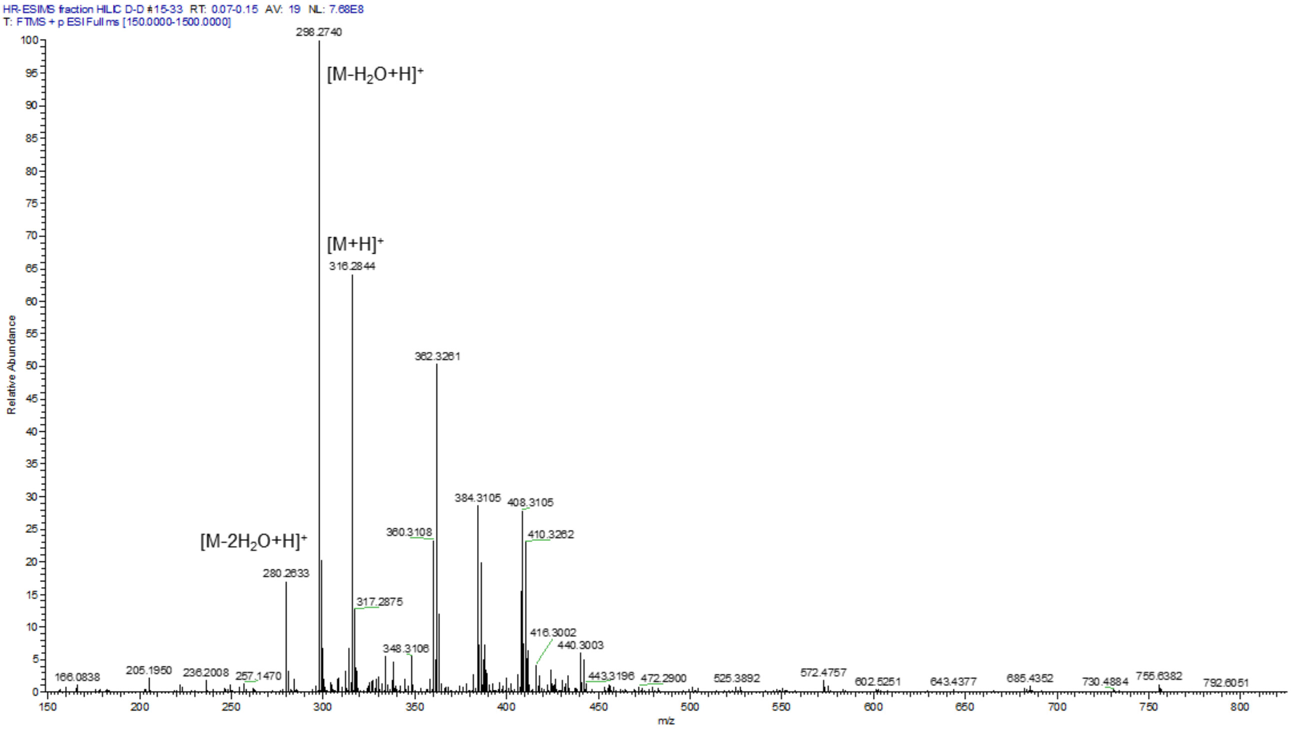

Figure S15. MSMS of the main compounds belonging to HILIC fraction D: A) MSMS spectrum of the ion peak of halisphingosine A at m/z 316.2844; B) MSMS spectrum of the ion peak at m/z 360.3108; C) MSMS spectrum of the ion peak at m/z 362.3261; D) MSMS spectrum of the ion peak at m/z 384.3105; E) MSMS spectrum of the ion peak at m/z 386.3262; F) MSMS spectrum of the ion peak at m/z 408.3105; G) MSMS spectrum of the ion peak at m/z 410.3262.

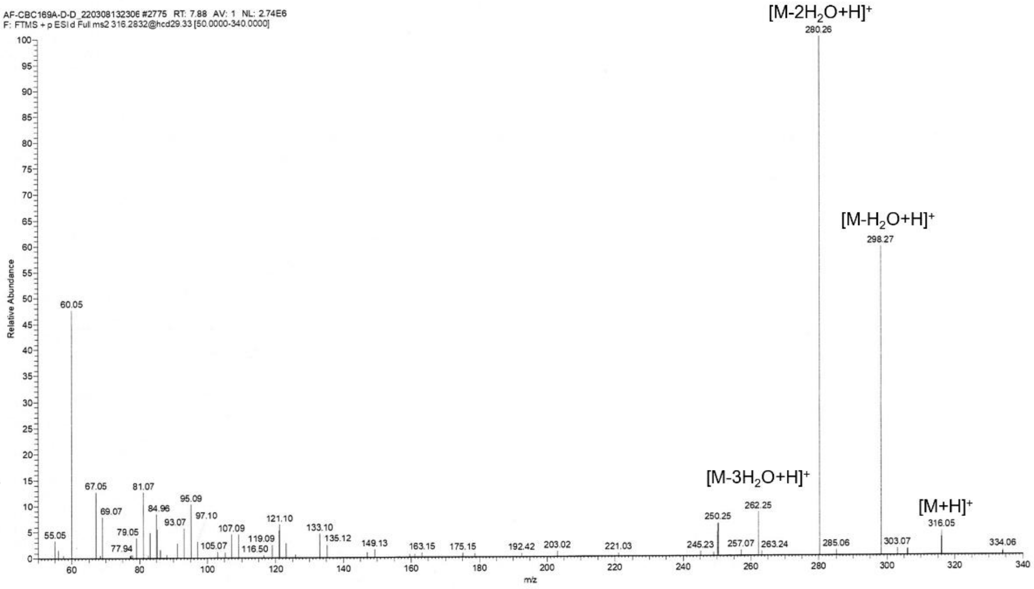

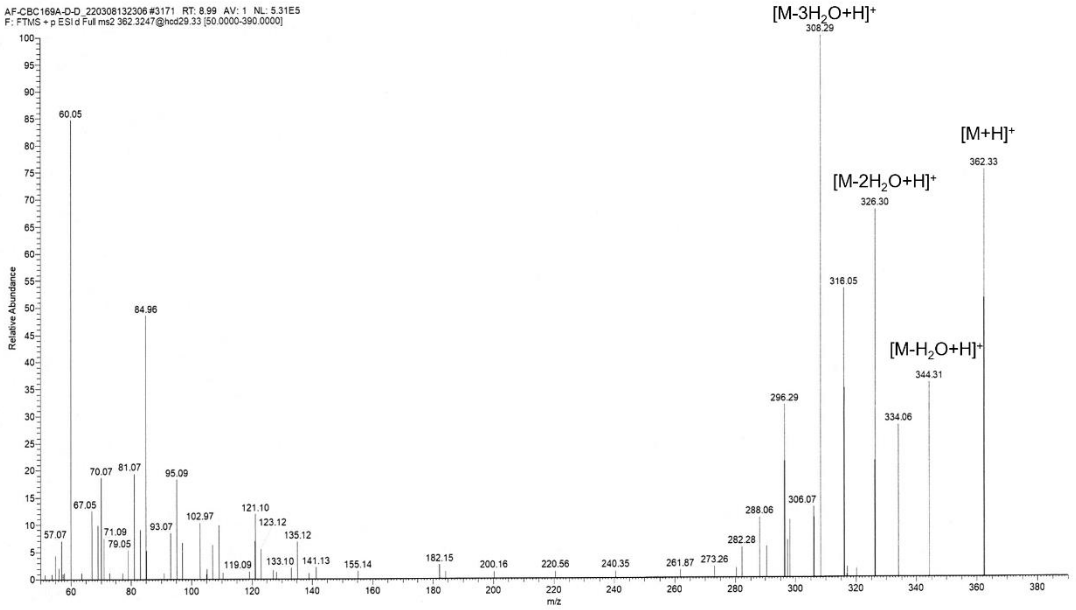
AF-CBC169A-D-D_220308132306 #3171 RT: 8.99 AV: 1 NL: 5.31E5
F: FTMS + p ESI d Full ms2 362.3247@hcd29.33 [50.0000-390.0000]
[M-3H2O+H]+
[M+H]+
[M-2H2O+H]+
[M-H2O+H]+
Relative Abundance
m/z

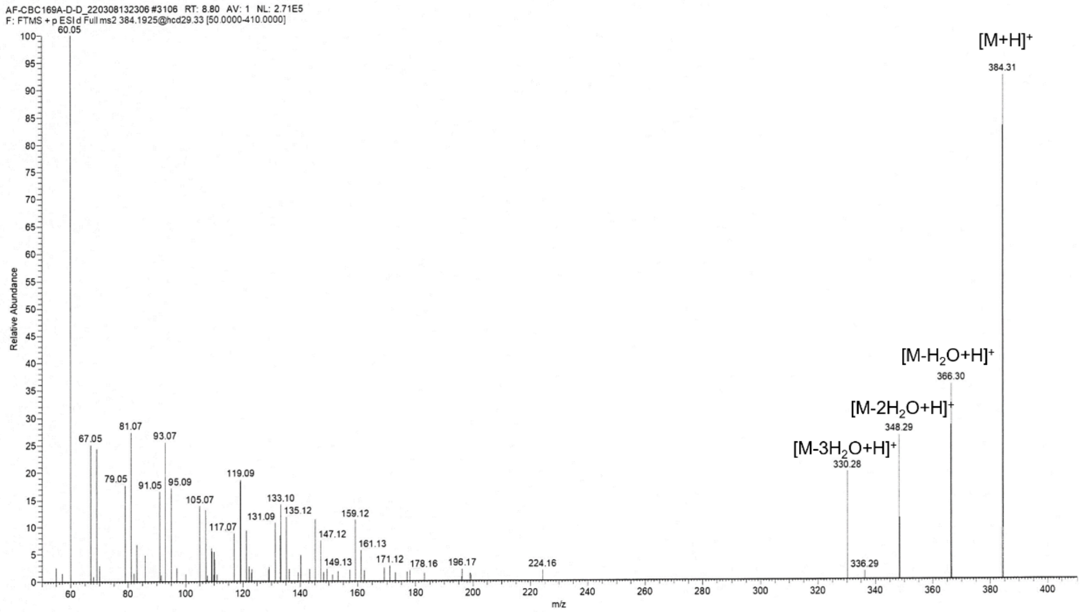
AF-CBC169A-D-D_220308132306 #3106 RT: 8.80 AV: 1 NL: 2.71E5
F: FTMS + p ESI d Full ms2 384.1925@hcd29.33 [50.0000-410.0000]
[M+H]+
[M-H2O+H]+
[M-2H2O+H]+
[M-3H2O+H]+
Relative Abundance
m/z

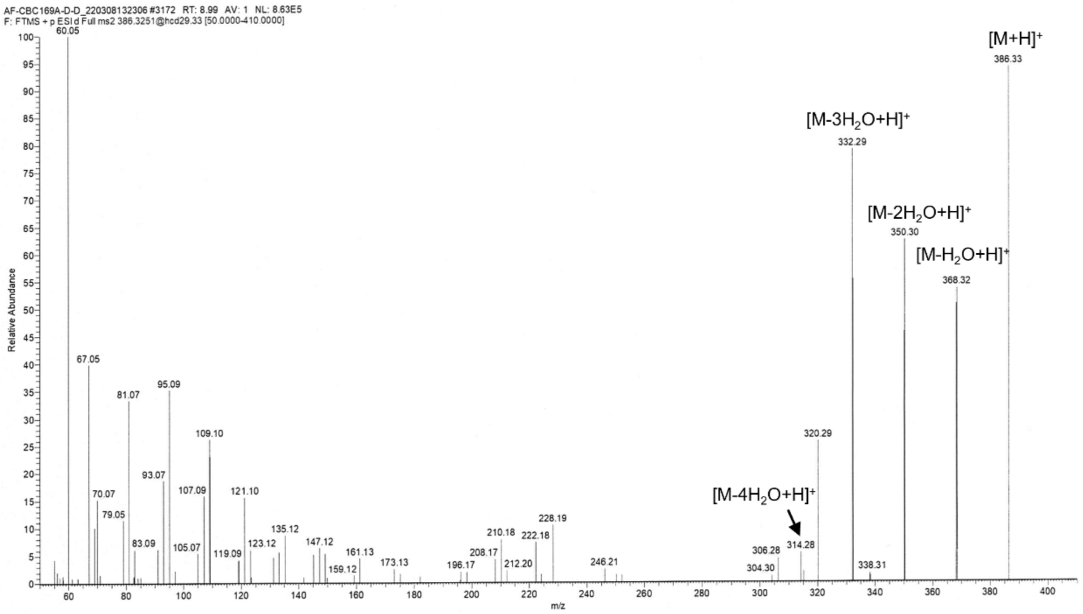
AF-CBC169A-D-D_220308132306 #3172 RT: 8.99 AV: 1 NL: 8.63E5
F: FTMS + p ESI d Full ms2 386.3251@hcd29.33 [50.0000-410.0000]
[M+H]+
[M-3H2O+H]+
[M-2H2O+H]+
[M-H2O+H]+
[M-4H2O+H]+
Relative Abundance
m/z

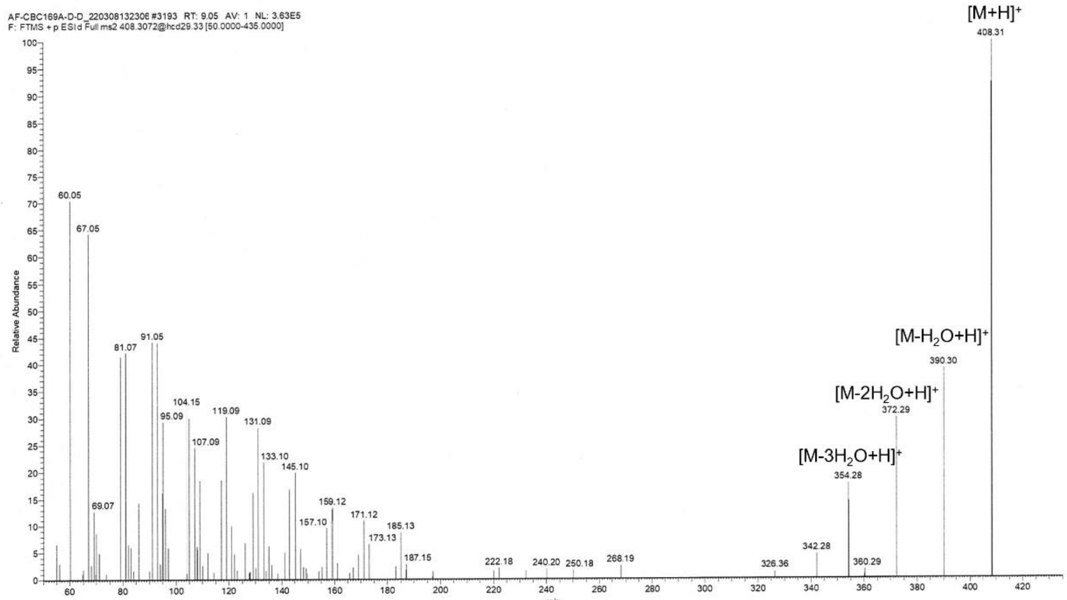
AF-CBC169A-D-D_220308132306 #3193 RT: 9.05 AV: 1 NL: 3.63E5
F: FTMS + p ESI d Full ms2 408.3072@hcd29.33 [50.0000-435.0000]
[M+H]+
[M-H2O+H]+
[M-2H2O+H]+
[M-3H2O+H]+
Relative Abundance
m/z

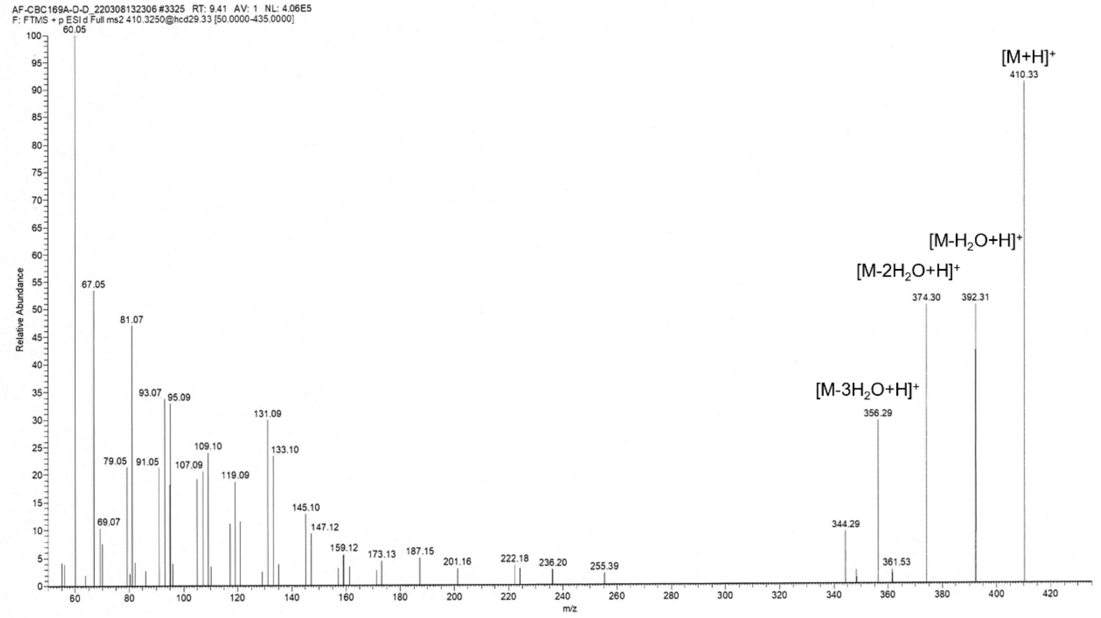
AF-CBC169A-D-D_220308132306 #3325 RT: 9.41 AV: 1 NL: 4.06E5
F: FTMS + p ESI d Full ms2 410.3250@hcd29.33 [50.0000-435.0000]
Relative Abundance
m/z
[M+H]+
[M-H2O+H]+
[M-2H2O+H]+
[M-3H2O+H]+

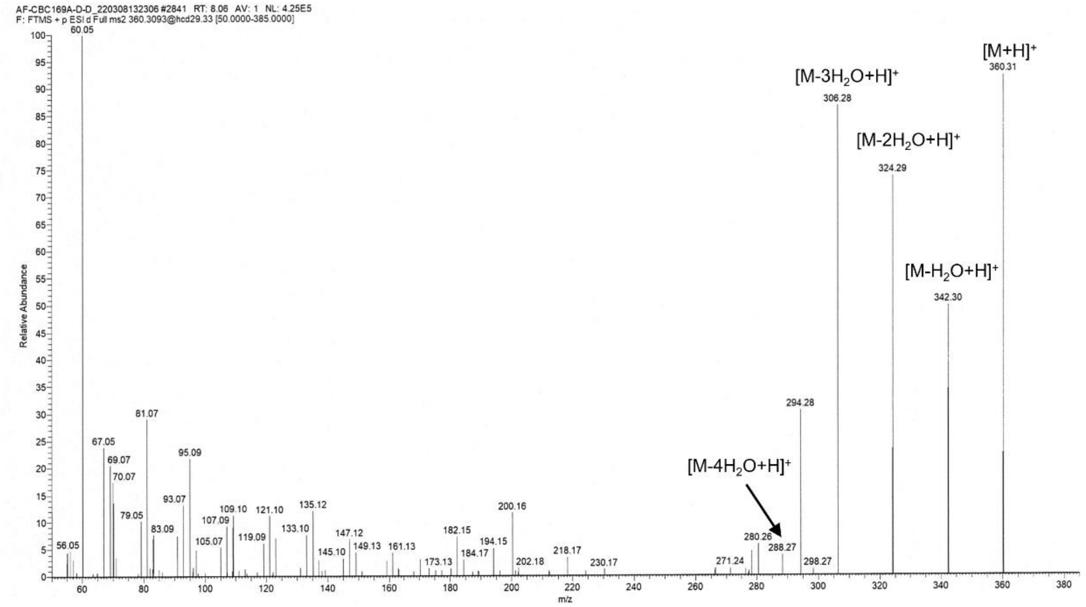
AF-CBC169A-D-D_220308132306 #2841 RT: 8.06 AV: 1 NL: 4.25E5
F: FTMS + p ESI d Full ms2 360.3093@hcd29.33 [50.0000-385.0000]
Relative Abundance
m/z
[M+H]+
[M-3H2O+H]+
[M-2H2O+H]+
[M-H2O+H]+
[M-4H2O+H]+

Figure S16. ^{1}H NMR of subfraction 1(red) and 2 (green) compared with sample C (blue) (600 MHz, CD_3OD).

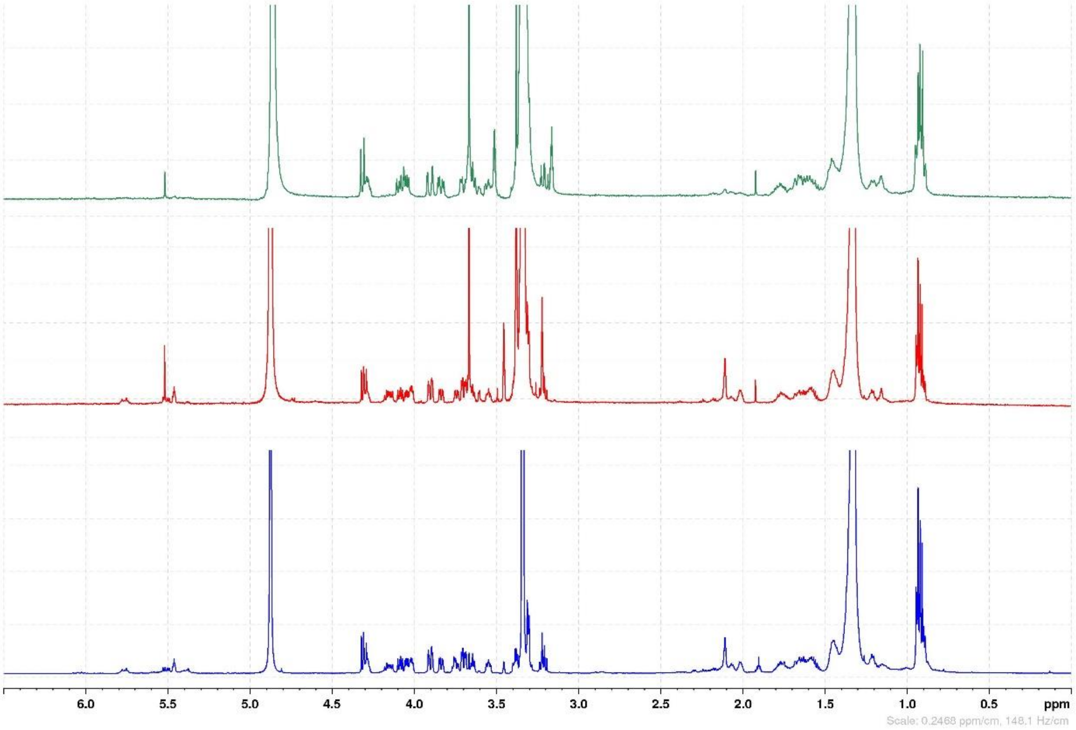

Figure S17. COSY NMR of subfraction 1 (600 MHz, CD_3OD).

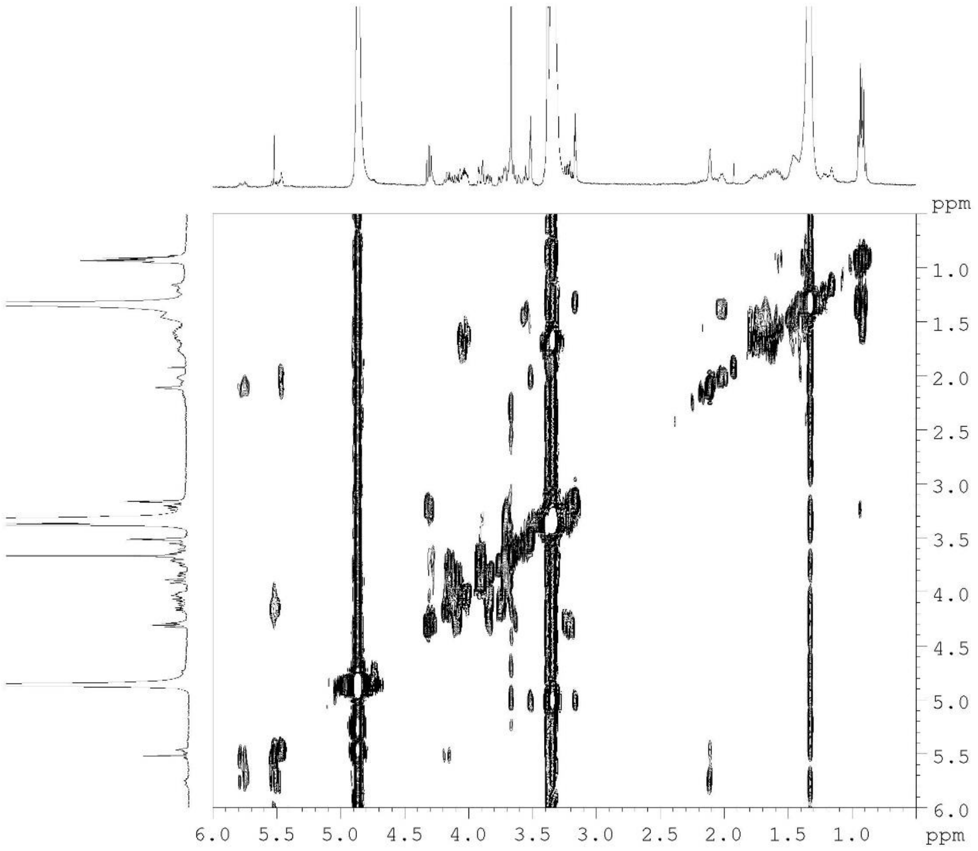

Figure S18. TOCSY NMR of subfraction 1 (600 MHz, CD_3OD).

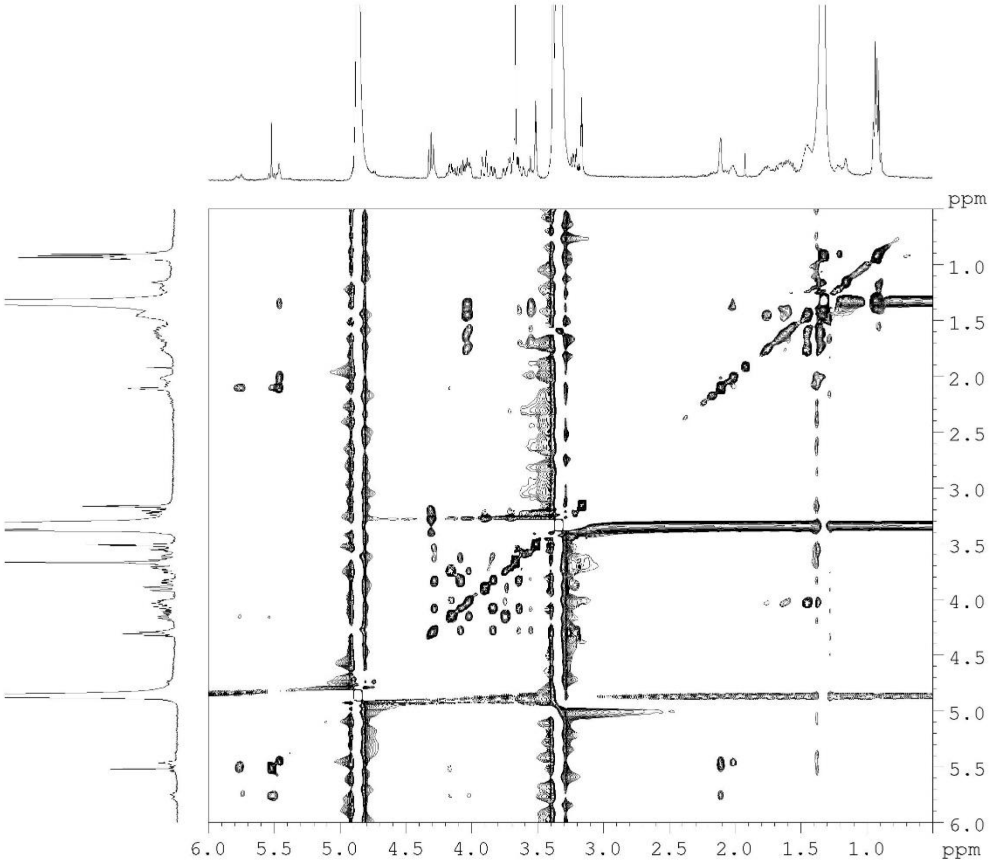

Figure S19. HSQC and HSQCed NMR (CH_2 are shown in light gray, while CH and CH_3 signals appear in black) of subfraction 1 (600 MHz, CD_3OD).

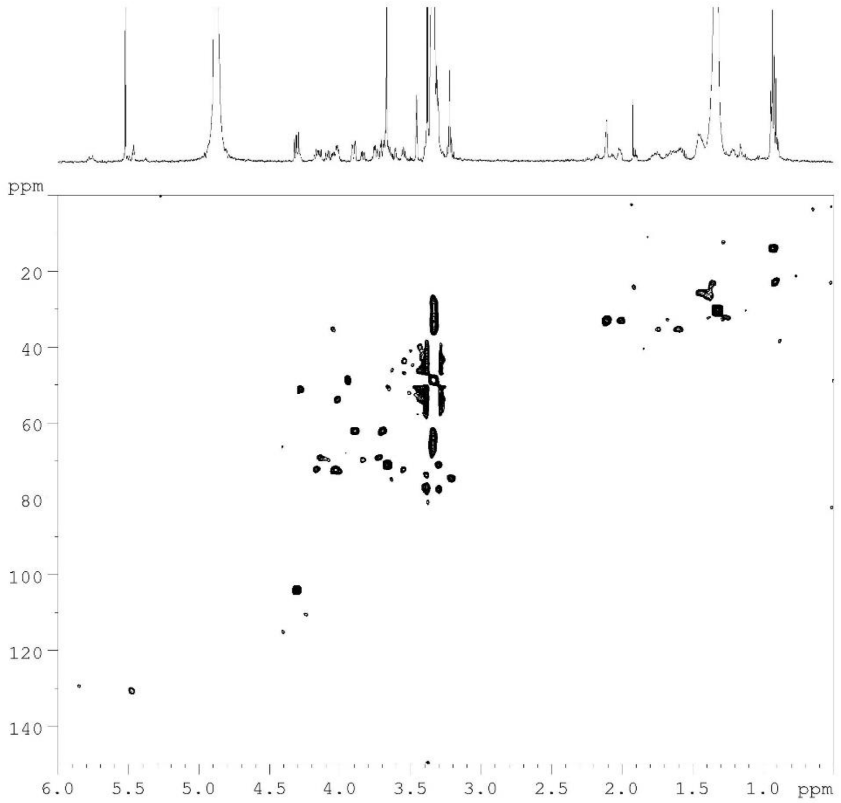

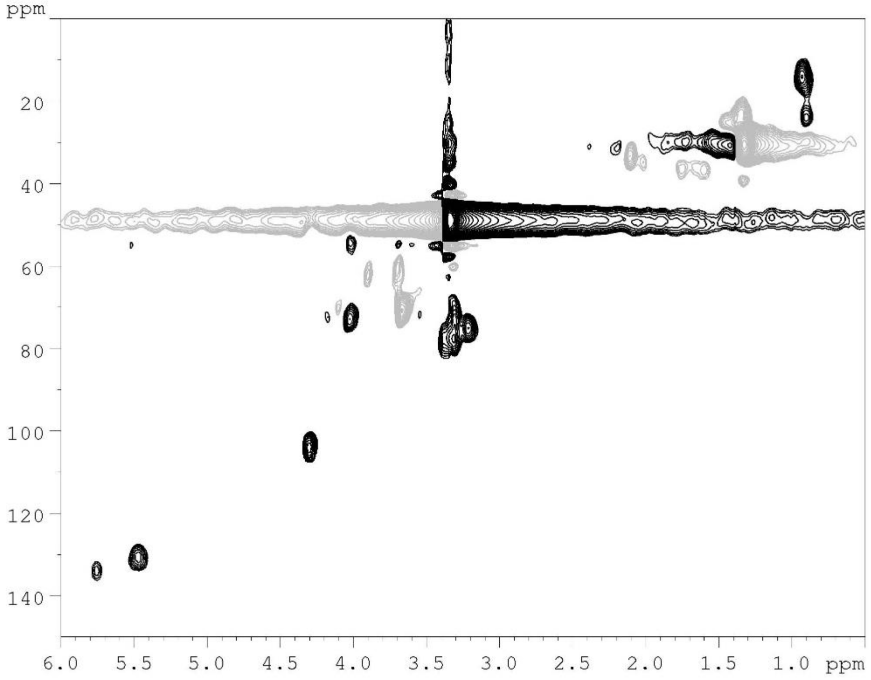

Figure S20. COSY NMR of subfraction 2 (600 MHz, CD_3OD).

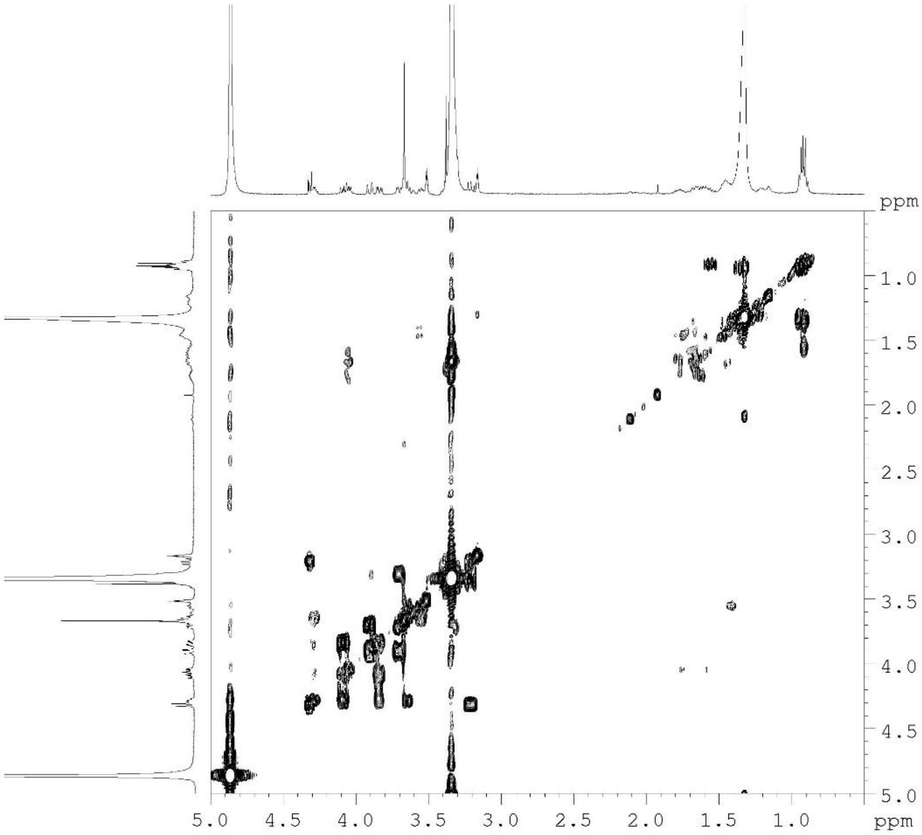

Figure S21. TOCSY NMR of subfraction 2 (600 MHz, CD_3OD).

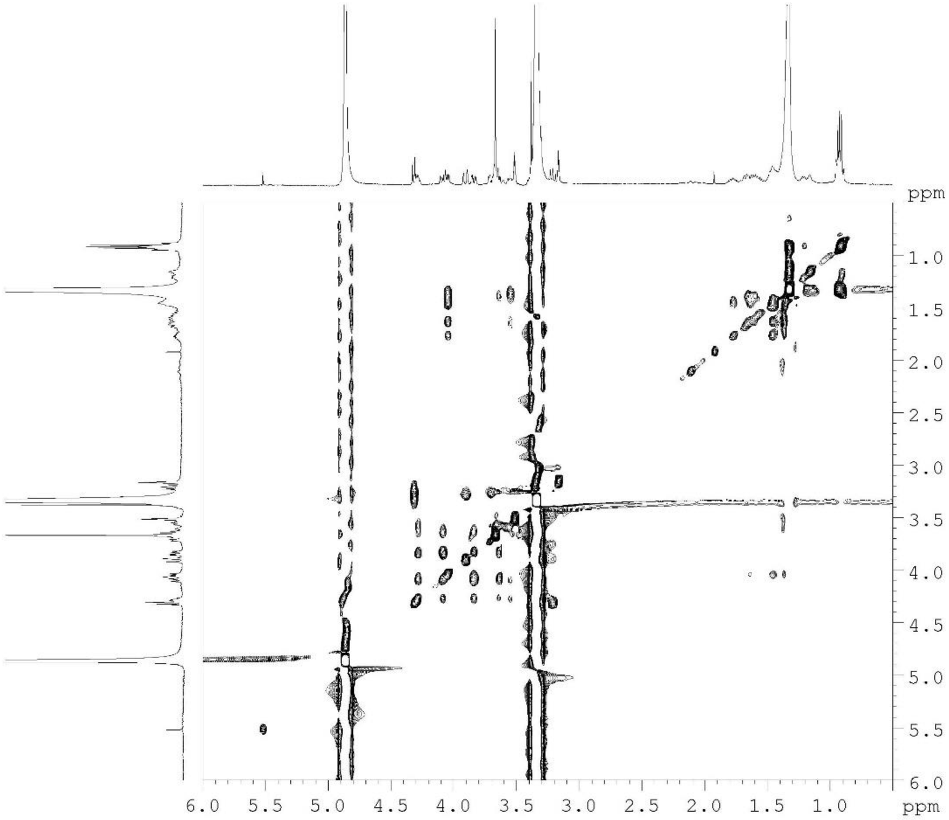

Figure S22. Full ESI⁻ MS of subfraction 1 and 2, compared with sample C.

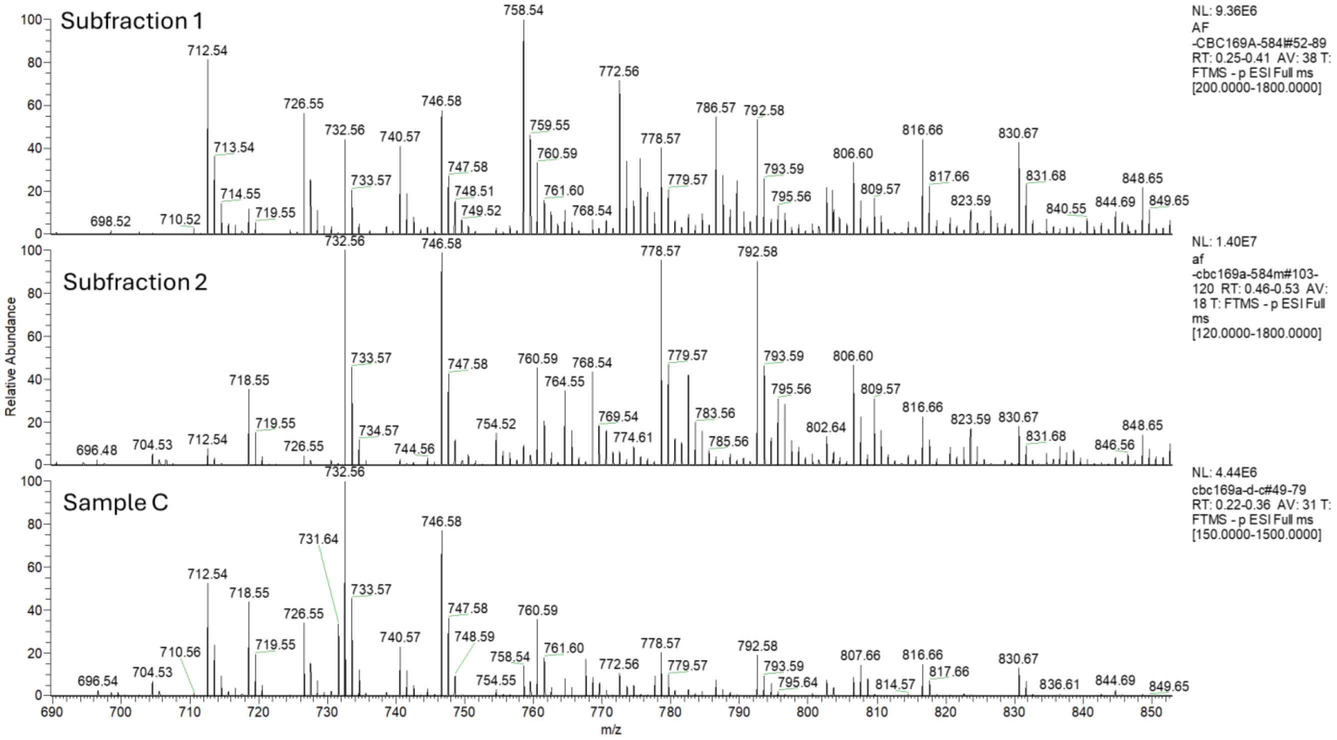

Figure S23. TLC of subfraction 1 and 2, compared with sample C.

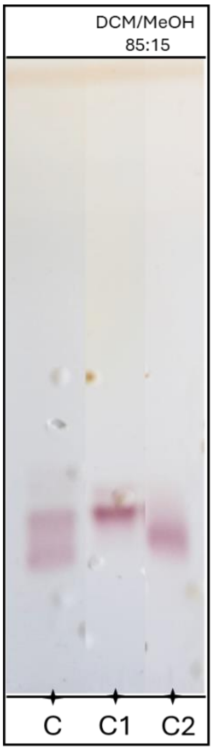

Figure S24. Immunoarray blots of the A2058 cells treated with the HILIC fraction D.

Relative expression of ten proteins involved in human angiogenesis in treated A2058 cells normalized vs control (untreated cells).

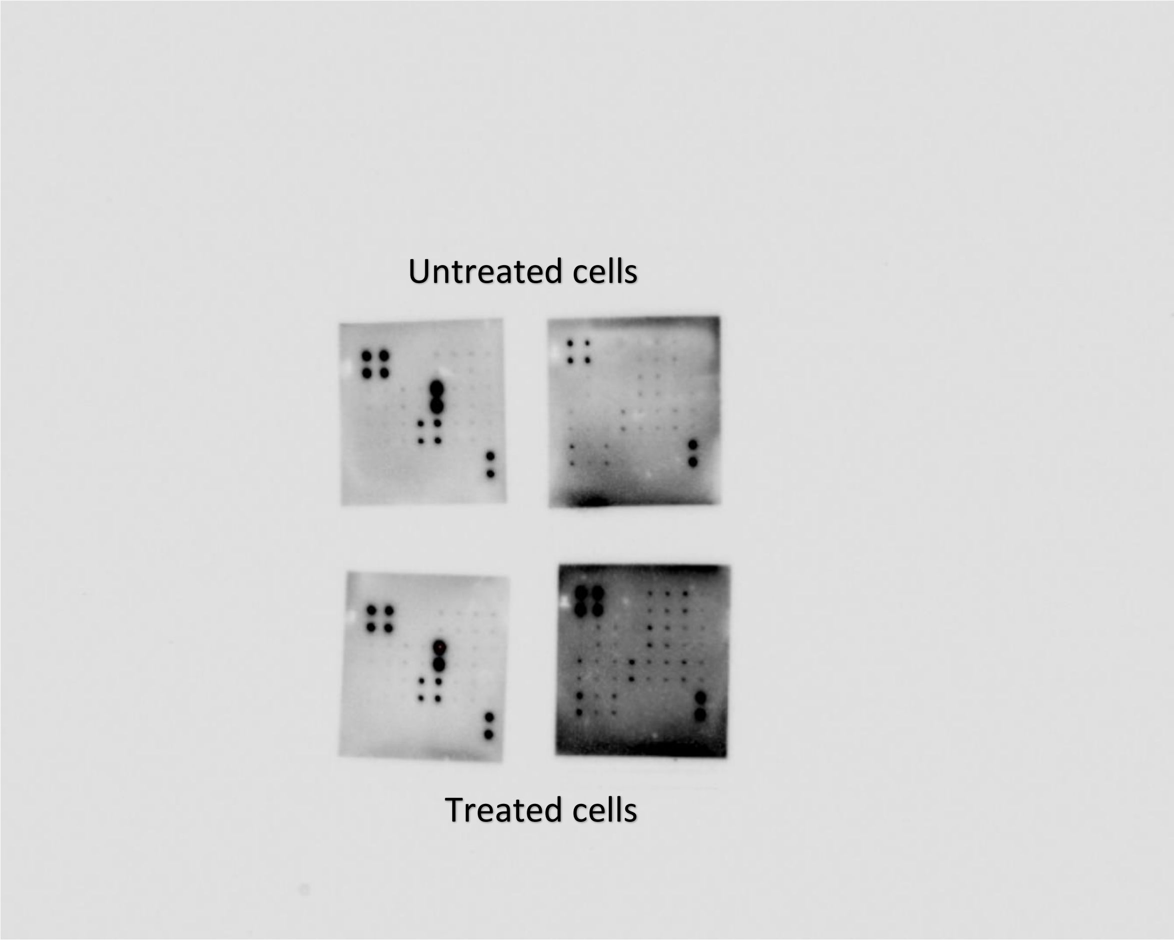

Table S1. Genes analyzed by PCR Array in A2058 cells after treatment with the HILIC fraction D. RT^2 Profiler PCR Arrays are highly reliable and sensitive gene expression profiling tools for analyzing focused panels of genes in signal transduction, biological processes or disease research pathways using real-time PCR. Each cataloged RT^2 Profiler PCR Array contains a list of the pathway-focused genes as well as five housekeeping (reference) genes on the array. In addition, each array contains a panel of proprietary controls to monitor genomic DNA contamination (GDC) as well as the first strand synthesis (RTC) and real-time PCR efficiency (PPC). The qPCR Assays used in PCR Arrays are laboratory-verified and optimized to work under standard conditions enabling many genes to be assayed simultaneously. Their specificity is guaranteed when RT^2 SYBR Green qPCR Master Mixes are used as part of the complete PCR Array System protocol.
In this study, 96 genes were profiled on 8 samples with the PAHS-178Z.

Position	Unigene	Refseq	Symbol	Description	Gname	RT2 Catalog
A01	Hs.157078	NM_144567	ANGEL2	Angel homolog 2 (Drosophila)	Ccr4d/KIAA0759L	PPH13038A
A02	Hs.480042	NM_005139	ANXA3	Annexin A3	ANX3	PPH06934F
A03	Hs.480653	NM_001154	ANXA5	Annexin A5	ANX5/ENX2/HEL-S-7/PP4/RPRGL3	PPH00304F
A04	Hs.468972	NM_006015	ARID1A	AT rich interactive domain 1A (SWI-like)	B120/BAF250/BAF250a	PPH13453B
A05	Hs.643580	NM_152522	ARL6IP6	ADP-ribosylation-like factor 6 interacting protein 6	AIP-6/PFAAP1	PPH12418A
A06	Hs.513645	NM_001211	BUB1B	Budding uninhibited by benzimidazoles 1 homolog beta (yeast)	BUB1beta/BUBR1/Bub1A/MAD3L/MVA1/SSK1/hBUBR1	PPH09047A
A07	Hs.632379	NM_015991	C1QA	Complement component 1, q subcomponent, A chain	-	PPH07701A
A08	Hs.8986	NM_000491	C1QB	Complement component 1, q subcomponent, B chain	-	PPH07660B
A09	Hs.467753	NM_172369	C1QC	Complement component 1, q subcomponent, C chain	C1Q-C/C1QG	PPH10562A
A10	Hs.458355	NM_001734	C1S	Complement component 1, s subcomponent	-	PPH22245A
A11	Hs.529053	NM_000064	C3	Complement component 3	AHUS5/ARMD9/ASP/C3a/C3b/CPAMD1/HEL-S-62p	PPH01185E
A12	Hs.591148	NM_004054	C3AR1	Complement component 3a receptor 1	AZ3B/C3AR/HNFAG09	PPH02514A
B01	Hs.2161	NM_001736	C5AR1	Complement component 5a receptor 1	C5A/C5AR/C5R1/CD88	PPH06063F
B02	Hs.65425	NM_004929	CALB1	Calbindin 1, 28kDa	CALB/D-28K	PPH02074A

B03	Hs.2490	NM_033292	CASP1	Caspase 1, apoptosis-related cysteine peptidase (interleukin 1, beta, convertase)	ICE/IL1BC/P45	PPH00105C
B04	Hs.301921	NM_001295	CCR1	Chemokine (C-C motif) receptor 1	CD191/CKR-1/CKR1/CMKBR1/HM145/MIP1aR/SCYAR1	PPH00611F
B05	Hs.163867	NM_000591	CD14	CD14 molecule	-	PPH05723A
B06	Hs.504641	NM_004244	CD163	CD163 molecule	M130/MM130	PPH05722A
B07	Hs.106070	NM_000076	CDKN1C	Cyclin-dependent kinase inhibitor 1C (p57, Kip2)	BWCR/BWS/KIP2/WBS/p57/p57Kip2	PPH00214B
B08	Hs.363396	NM_000186	CFH	Complement factor H	AHUS1/AMBP1/ARMD4/ARMS1/CFHL3/FH/FHL1/HF/HF1/HF2/HUS	PPH15684E
B09	Hs.436657	NM_001831	CLU	Clusterin	AAG4/APO-J/APOJ/CLI/CLU1/CLU2/KUB1/NA1/NA2/SGP-2/SGP2/SP-40/TRPM-2/TRPM2	PPH00243F
B10	Hs.172928	NM_000088	COL1A1	Collagen, type I, alpha 1	EDSC/OI1/OI2/OI3/OI4	PPH01299F
B11	Hs.443625	NM_000090	COL3A1	Collagen, type III, alpha 1	EDS4A	PPH00439F
B12	Hs.531668	NM_002996	CX3CL1	Chemokine (C-X3-C motif) ligand 1	ABCD-3/C3Xkine/CXC3/CXC3C/NTN/NTT/SCYD1/fractalkine/neurotactin	PPH00689C
C01	Hs.745037	NM_022059	CXCL16	Chemokine (C-X-C motif) ligand 16	CXCLG16/SR-PSOX/SRPSOX	PPH01344A
C02	Hs.184492	NM_001419	ELAVL1	ELAV (embryonic lethal, abnormal vision, Drosophila)-like 1 (Hu antigen R)	ELAV1/HUR/Hua/MelG	PPH13883B
C03	Hs.491336	NM_018091	ELP3	Elongation protein 3 homolog (S. cerevisiae)	KAT9	PPH15366A
C04	Hs.12451	NM_004434	EML1	Echinoderm microtubule associated protein like 1	ELP79/EMAP/EMAPL/HuEMAP	PPH07860A
C05	Hs.517517	NM_001429	EP300	E1A binding protein p300	KAT3B/RSTS2/p300	PPH00319A
C06	Hs.513244	NM_153350	FBXL16	F-box and leucine-rich repeat protein 16	C16orf22/Fbl16/c380A1.1	PPH21217B
C07	Hs.433300	NM_004106	FCER1G	Fc fragment of IgE, high affinity I, receptor for; gamma polypeptide	FCRG	PPH02628B
C08	Hs.111732	NM_003890	FCGBP	Fc fragment of IgG binding protein	FC(GAMMA)BP	PPH11190A
C09	Hs.77424	NM_000566	FCGR1A	Fc fragment of IgG, high affinity Ia, receptor (CD64)	CD64/CD64A/FCRI/IGFR1	PPH00858A
C10	Hs.352642	NM_021642	FCGR2A	Fc fragment of IgG, low affinity IIa, receptor (CD32)	CD32/CD32A/CDw32/FCG2/FCGR2/FCGR2A1/FcGR/IGFR2	PPH17649B

C11	Hs.694258	NM_000570	FCGR3B	Fc fragment of IgG, low affinity IIIb, receptor (CD16b)	CD16/CD16b/FCG3/FCGR3/FCR-10/FCRIII/FCRIIIb	PPH01487G
C12	Hs.370666	NM_002015	FOXO1	Forkhead box O1	FKH1/FKHR/FOXO1A	PPH01964F
D01	Hs.514227	NM_002055	GFAP	Glial fibrillary acidic protein	ALXDRD	PPH02408F
D02	Hs.446309	NM_145740	GSTA1	Glutathione S-transferase alpha 1	GST2/GSTA1-1/GTH1	PPH00311B
D03	Hs.530227	NM_005526	HSF1	Heat shock transcription factor 1	HSTF1	PPH00164F
D04	Hs.106254	NM_001105521	JAKMIP3	Janus kinase and microtubule interacting protein 3	C10orf14/C10orf39/Jamip3/NECC2/bA140A10.5	PPH11023A
D05	Hs.594444	NM_005572	LMNA	Lamin A/C	CDCD1/CDDC/CMD1A/CMT2B1/EMD2/FPL/FPLD/FPLD2/HGPS/IDC/LDP1/LFP/LGMD1B/LMN1/LMNC/LMNL1/PRO1	PPH20587F
D06	Hs.89497	NM_005573	LMNB1	Lamin B1	ADLD/LMN/LMN2/LMNB	PPH00278B
D07	Hs.538286	NM_032737	LMNB2	Lamin B2	LAMB2/LMN2	PPH10007A
D08	Hs.424908	NM_012322	LSM5	LSM5 homolog, U6 small nuclear RNA associated (S. cerevisiae)	YER146W	PPH19971A
D09	Hs.529517	NM_002343	LTF	Lactotransferrin	GIG12/HEL110/HLF2/LF	PPH05751A
D10	Hs.524579	NM_000239	LYZ	Lysozyme	LZM	PPH14748A
D11	Hs.551713	NM_002385	MBP	Myelin basic protein	-	PPH02627A
D12	Hs.421848	NM_176794	MRPL43	Mitochondrial ribosomal protein L43	L43mt/MRP-L43/bMRP36a	PPH19909A
E01	Hs.521969	NM_019056	NDUFB11	NADH dehydrogenase (ubiquinone) 1 beta subcomplex, 11, 17.3kDa	CI-ESSS/ESSS/LSDMCA3/NP17.3/Np15/P17.3	PPH20172A
E02	Hs.591976	NM_015368	PANX1	Pannexin 1	MRS1/PX1/UNQ2529	PPH08954A
E03	Hs.50823	NM_013232	PDCD6	Programmed cell death 6	ALG-2/ALG2/PEF1B	PPH00068F
E04	Hs.731957	NM_015153	PHF3	PHD finger protein 3	-	PPH17417A
E05	Hs.254113	NM_005035	POLRMT	Polymerase (RNA) mitochondrial (DNA directed)	APOLMT/MTRNAP/MTRPOL/h-mtRPOL	PPH15163E
E06	Hs.31968	NM_015450	POT1	Protection of telomeres 1 homolog (S. pombe)	CMM10/HPOT1	PPH08949F

E07	Hs.586618	NM_002884	RAP1A	RAP1A, member of RAS oncogene family	C21KG/G-22K/KREV-1/KREV1/RAP1/SMGP21	PPH02284F
E08	Hs.148741	NM_182757	RNF144B	Ring finger protein 144B	IBRDC2/PIR2/bA528A10.3/p53RFP	PPH20115B
E09	Hs.416073	NM_002964	S100A8	S100 calcium binding protein A8	60B8AG/CAGA/CFAG/CGLA/CP-10/L1Ag/MA387/MIF/MRP8/NIF/P8	PPH19755A
E10	Hs.112405	NM_002965	S100A9	S100 calcium binding protein A9	60B8AG/CAGB/CFAG/CGLB/L1AG/LIAG/MAC387/MIF/MRP14/NIF/P14	PPH11215E
E11	Hs.129783	NM_004588	SCN2B	Sodium channel, voltage-gated, type II, beta	ATFB14	PPH01430A
E12	Hs.369779	NM_012238	SIRT1	Sirtuin 1	SIR2/SIR2L1/SIR2alpha	PPH02188A
F01	Hs.716456	NM_012239	SIRT3	Sirtuin 3	SIR2L3	PPH22989A
F02	Hs.423756	NM_016539	SIRT6	Sirtuin 6	SIR2L6	PPH19944A
F03	Hs.12253	NM_005901	SMAD2	SMAD family member 2	JV18/JV18-1/MADH2/MADR2/hMAD-2/hSMAD2	PPH01949F
F04	Hs.511149	NM_003825	SNAP23	Synaptosomal-associated protein, 23kDa	HsT17016/SNAP-23/SNAP23A/SNAP23B	PPH00761B
F05	Hs.442707	NM_017489	TERF1	Telomeric repeat binding factor (NIMA-interacting) 1	PIN2/TRBF1/TRF/TRF1/hTRF1-AS/t-TRF1	PPH02426A
F06	Hs.63335	NM_005652	TERF2	Telomeric repeat binding factor 2	TRBF2/TRF2	PPH02738A
F07	Hs.594250	NM_003201	TFAM	Transcription factor A, mitochondrial	MTTF1/MTTFA/TCF6/TCF6L1/TCF6L2/TCF6L3	PPH09934B
F08	Hs.279908	NM_016020	TFB1M	Transcription factor B1, mitochondrial	CGI-75/CGI75/mtTFB/mtTFB1	PPH15798C
F09	Hs.7395	NM_022366	TFB2M	Transcription factor B2, mitochondrial	Hkp1/mtTFB2	PPH07510A
F10	Hs.496191	NM_012461	TINF2	TERF1 (TRF1)-interacting nuclear factor 2	DKCA3/TIN2	PPH02468A
F11	Hs.519033	NM_003264	TLR2	Toll-like receptor 2	CD282/TIL4	PPH01808A
F12	Hs.174312	NM_138554	TLR4	Toll-like receptor 4	ARMD10/CD284/TLR-4/TOLL	PPH01795F
G01	Hs.188591	NM_022918	TMEM135	Transmembrane protein 135	PMP52	PPH13867A
G02	Hs.31082	NM_018126	TMEM33	Transmembrane protein 33	1600019D15Rik/SHINC3	PPH08910A

G03	Hs.368527	NM_019009	TOLLIP	Toll interacting protein	IL-1RAcPIP	PPH05844C
G04	Hs.523454	NM_000391	TPP1	Tripeptidyl peptidase I	CLN2/GIG1/LPIC/SCAR7/TPP-1	PPH20033B
G05	Hs.533977	NM_006472	TXNIP	Thioredoxin interacting protein	EST01027/HHCPA78/THIF/VDUP1	PPH02855A
G06	Hs.511668	NM_017684	VPS13C	Vacuolar protein sorting 13 homolog C (S. cerevisiae)	-	PPH17028A
G07	Hs.152944	NM_198315	VWA5A	Von Willebrand factor A domain containing 5A	BCSC-1/BCSC1/LOH11CR2A	PPH12864A
G08	Hs.632050	NM_000553	WRN	Werner syndrome, RecQ helicase-like	RECQ3/RECQL2/RECQL3	PPH02160F
G09	Hs.591868	NM_023929	ZBTB10	Zinc finger and BTB domain containing 10	RINZF	PPH20661F
G10	Hs.435231	NM_016107	ZFR	Zinc finger RNA binding protein	SPG71/ZFR1	PPH13591B
G11	Hs.132642	NM_005857	ZMPSTE24	Zinc metallopeptidase (STE24 homolog, S. cerevisiae)	FACE-1/FACE1/HGPS/PRO1/STE24/Ste24p	PPH08627A
G12	Hs.499429	NM_145011	ZNF25	Zinc finger protein 25	KOX19/Zfp9	PPH07362A
H01	Hs.520640	NM_001101	ACTB	Actin, beta	BRWS1/PS1TP5BP1	PPH00073G
H02	Hs.534255	NM_004048	B2M	Beta-2-microglobulin	-	PPH01094E
H03	Hs.592355	NM_002046	GAPDH	Glyceraldehyde-3-phosphate dehydrogenase	G3PD/GAPD/HEL-S-162eP	PPH00150F
H04	Hs.412707	NM_000194	HPRT1	Hypoxanthine phosphoribosyltransferase 1	HGPRT/HPRT	PPH01018C
H05	Hs.546285	NM_001002	RPLP0	Ribosomal protein, large, P0	L10E/LP0/P0/PRLP0/RPP0	PPH21138F
H06	N/A	SA_00105	HGDC	Human Genomic DNA Contamination	HIGX1A	
H07	N/A	SA_00104	RTC	Reverse Transcription Control	RTC	PPX63340A
H08	N/A	SA_00104	RTC	Reverse Transcription Control	RTC	PPX63340A
H09	N/A	SA_00104	RTC	Reverse Transcription Control	RTC	PPX63340A
H10	N/A	SA_00103	PPC	Positive PCR Control	PPC	
H11	N/A	SA_00103	PPC	Positive PCR Control	PPC	
H12	N/A	SA_00103	PPC	Positive PCR Control	PPC	

Table S2. Fold-Change (2^(-Delta Delta CT)) is the normalized gene expression (2^(- Delta CT)) in the Test Sample divided the normalized gene expression (2^(- Delta CT)) in the Control Sample in A2058 cells. Fold-Regulation represents fold-change results in a biologically meaningful way. Fold-change values greater than one indicates a positive- or an up-regulation, and the fold-regulation is equal to the fold-change. Fold-change values less than one indicate a negative or down-regulation, and the fold-regulation is the negative inverse of the fold-change.

Position	Gene Symbol	Fold Regulation
A01	ANGEL2	2.14
A02	ANXA3	348.01
A03	ANXA5	4.05
A06	BUB1B	71.15
A07	C1QA	32.70
A08	C1QB	16.03
A09	C1QC	33.22
A10	C1S	3.45
A11	C3	5.73
A12	C3AR1	2.06
B01	C5AR1	2.82
B03	CASP1	63.58
B04	CCR1	42.09
B05	CD14	45.20
B06	CD163	276.41
B07	CDKN1C	96.37
B08	CFH	11.39
B10	COL1A1	13.76
B11	COL3A1	46.03
C02	ELAVL1	8.43
C04	EML1	40.15
C06	FBXL16	13.50
C08	FCGBP	46.03
C09	FCGR1A	36.27
C10	FCGR2A	91.02
C11	FCGR3B	46.03
D01	GFAP	21.93
D02	GSTA1	46.03
D04	JAKMIP3	6.72
D06	LMNB1	10.72
D08	LSM5	63.60
D09	LTF	46.03
D11	MBP	11.42
D12	MRPL43	2.86
E03	PDCD6	3.24
E05	POLRMT	6.85
E06	POT1	5.94
E07	RAP1A	8.00
E08	RNF144B	58.68
E09	S100A8	46.03
E10	S100A9	32.71
E11	SCN2B	46.03
E12	SIRT1	3.48
F03	SMAD2	2.50
F04	SNAP23	98.18
F07	TFAM	7.19
F08	TFB1M	7.21
F11	TLR2	46.03
G01	TMEM135	130.85
G02	TMEM33	6.45
G05	TXNIP	2.58
G06	VPS13C	4.76
G08	WRN	5.56
G09	ZBTB10	15.24
G11	ZMPSTE24	636.40
G12	ZNF25	10.32
H02	B2M	6.22
H04	HPRT1	3.20
H08	RPLP0	11.25
A05	ARL6IP6	-9.64
B09	CLU	-11.59
B12	CX3CL1	-4.28
C01	CXCL16	-3.50
C03	ELP3	-97.12
C12	FOXO1	-3.22
D05	LMNA	-6.42
E02	PANX1	-9.92
F01	SIRT3	-2.61
F02	SIRT6	-4.19
F05	TERF1	-5.27
F09	TFB2M	-21.20
F12	TLR4	-7.26
G04	TPP1	-8.06
G07	VWA5A	-2.27
G10	ZFR	-2.80
H01	ACTB	-11.42
H03	GAPDH	-19.60

Table S3. Genes analyzed by PCR Array in PNT2 cells after treatment with the HILIC fraction D. RT² Profiler PCR Arrays are highly reliable and sensitive gene expression profiling tools for analyzing focused panels of genes in signal transduction, biological processes or disease research pathways using real-time PCR. Each cataloged RT² Profiler PCR Array contains a list of the pathway-focused genes as well as five housekeeping (reference) genes on the array. In addition, each array contains a panel of proprietary controls to monitor genomic DNA contamination (GDC) as well as the first strand synthesis (RTC) and real-time PCR efficiency (PPC). The qPCR Assays used in PCR Arrays are laboratory-verified and optimized to work under standard conditions enabling

many genes to be assayed simultaneously. Their specificity is guaranteed when RT² SYBR Green qPCR Master Mixes are used as part of the complete PCR Array System protocol.
In this study, 96 genes were profiled on 8 samples with the PAHS-406Z.

Position	RefSeq Number	Symbol	Description
A01	NM_002986	CCL11	Chemokine (C-C motif) ligand 11
A02	NM_005408	CCL13	Chemokine (C-C motif) ligand 13
A03	NM_004590	CCL16	Chemokine (C-C motif) ligand 16
A04	NM_006274	CCL19	Chemokine (C-C motif) ligand 19
A05	NM_002982	CCL2	Chemokine (C-C motif) ligand 2
A06	NM_002983	CCL3	Chemokine (C-C motif) ligand 3
A07	NM_002985	CCL5	Chemokine (C-C motif) ligand 5
A08	NM_006273	CCL7	Chemokine (C-C motif) ligand 7
A09	NM_005623	CCL8	Chemokine (C-C motif) ligand 8
A10	NM_001295	CCR1	Chemokine (C-C motif) receptor 1
A11	NM_001123396	CCR2	Chemokine (C-C motif) receptor 2
A12	NM_001837	CCR3	Chemokine (C-C motif) receptor 3
B01	NM_000579	CCR5	Chemokine (C-C motif) receptor 5
B02	NM_001763	CD1A	CD1a molecule
B03	NM_001764	CD1B	CD1b molecule
B04	NM_001765	CD1C	CD1c molecule
B05	NM_001766	CD1D	CD1d molecule
B06	NM_001767	CD2	CD2 molecule
B07	NM_021155	CD209	CD209 molecule
B08	NM_006139	CD28	CD28 molecule
B09	NM_000616	CD4	CD4 molecule
B10	NM_001250	CD40	CD40 molecule, TNF receptor superfamily member 5
B11	NM_000074	CD40LG	CD40 ligand
B12	NM_000610	CD44	CD44 molecule (Indian blood group)
C01	NM_004355	CD74	CD74 molecule, major histocompatibility complex, class II invariant chain
C02	NM_005191	CD80	CD80 molecule
C03	NM_006889	CD86	CD86 molecule
C04	NM_001768	CD8A	CD8a molecule
C05	NM_001791	CDC42	Cell division cycle 42 (GTP binding protein, 25kDa)
C06	NM_000389	CDKN1A	Cyclin-dependent kinase inhibitor 1A (p21, Cip1)
C07	NM_004364	CEBPA	CCAAT/enhancer binding protein (C/EBP), alpha
C08	NM_130441	CLEC4C	C-type lectin domain family 4, member C
C09	NM_005211	CSF1R	Colony stimulating factor 1 receptor
C10	NM_000758	CSF2	Colony stimulating factor 2 (granulocyte-macrophage)

Position	RefSeq Number	Symbol	Description
C11	NM_001511	CXCL1	Chemokine (C-X-C motif) ligand 1 (melanoma growth stimulating activity, alpha)
C12	NM_001565	CXCL10	Chemokine (C-X-C motif) ligand 10
D01	NM_000609	CXCL12	Chemokine (C-X-C motif) ligand 12
D02	NM_002089	CXCL2	Chemokine (C-X-C motif) ligand 2
D03	NM_000634	CXCR1	Chemokine (C-X-C motif) receptor 1
D04	NM_003467	CXCR4	Chemokine (C-X-C motif) receptor 4
D05	NM_004448	ERBB2	V-erb-b2 erythroblastic leukemia viral oncogene homolog 2, neuro/glioblastoma derived oncogene homolog (avian)
D06	NM_000043	FAS	Fas (TNF receptor superfamily, member 6)
D07	NM_002001	FCER1A	Fc fragment of IgE, high affinity I, receptor for; alpha polypeptide
D08	NM_002002	FCER2	Fc fragment of IgE, low affinity II, receptor for (CD23)
D09	NM_000566	FCGR1A	Fc fragment of IgG, high affinity Ia, receptor (CD64)
D10	NM_004119	FLT3	Fms-related tyrosine kinase 3
D11	NM_001459	FLT3LG	Fms-related tyrosine kinase 3 ligand
D12	NM_002116	HLA-A	Major histocompatibility complex, class I, A
E01	NM_006120	HLA-DMA	Major histocompatibility complex, class II, DM alpha
E02	NM_033554	HLA-DPA1	Major histocompatibility complex, class II, DP alpha 1
E03	NM_000201	ICAM1	Intercellular adhesion molecule 1
E04	NM_000873	ICAM2	Intercellular adhesion molecule 2
E05	NM_000619	IFNG	Interferon, gamma
E06	NM_000572	IL10	Interleukin 10
E07	NM_000882	IL12A	Interleukin 12A (natural killer cell stimulatory factor 1, cytotoxic lymphocyte maturation factor 1, p35)
E08	NM_002187	IL12B	Interleukin 12B (natural killer cell stimulatory factor 2, cytotoxic lymphocyte maturation factor 2, p40)
E09	NM_004513	IL16	Interleukin 16
E10	NM_000586	IL2	Interleukin 2
E11	NM_000600	IL6	Interleukin 6 (interferon, beta 2)
E12	NM_000584	CXCL8	Interleukin 8
F01	NM_001572	IRF7	Interferon regulatory factor 7
F02	NM_002163	IRF8	Interferon regulatory factor 8
F03	NM_000632	ITGAM	Integrin, alpha M (complement component 3 receptor 3 subunit)
F04	NM_000211	ITGB2	Integrin, beta 2 (complement component 3 receptor 3 and 4 subunit)
F05	NM_002332	LRP1	Low density lipoprotein receptor-related protein 1
F06	NM_002350	LYN	V-yes-1 Yamaguchi sarcoma viral related oncogene homolog
F07	NM_002415	MIF	Macrophage migration inhibitory factor (glycosylation-inhibiting factor)
F08	NM_003998	NFKB1	Nuclear factor of kappa light polypeptide gene enhancer in B-cells 1
F09	NM_002838	PTPRC	Protein tyrosine phosphatase, receptor type, C
F10	NM_006908	RAC1	Ras-related C3 botulinum toxin substrate 1 (rho family, small GTP binding protein Rac1)
F11	NM_021975	RELA	V-rel reticuloendotheliosis viral oncogene homolog A (avian)

Position	RefSeq Number	Symbol	Description
F12	NM_006509	RELB	V-rel reticuloendotheliosis viral oncogene homolog B
G01	NM_003150	STAT3	Signal transducer and activator of transcription 3 (acute-phase response factor)
G02	NM_000544	TAP2	Transporter 2, ATP-binding cassette, sub-family B (MDR/TAP)
G03	NM_003190	TAPBP	TAP binding protein (tapasin)
G04	NM_000660	TGFB1	Transforming growth factor, beta 1
G05	NM_003246	THBS1	Thrombospondin 1
G06	NM_003263	TLR1	Toll-like receptor 1
G07	NM_003264	TLR2	Toll-like receptor 2
G08	NM_016562	TLR7	Toll-like receptor 7
G09	NM_017442	TLR9	Toll-like receptor 9
G10	NM_000594	TNF	Tumor necrosis factor
G11	NM_003701	TNFSF11	Tumor necrosis factor (ligand) superfamily, member 11
G12	NM_001078	VCAM1	Vascular cell adhesion molecule 1
H01	NM_001101	ACTB	Actin, beta
H02	NM_004048	B2M	Beta-2-microglobulin
H03	NM_002046	GAPDH	Glyceraldehyde-3-phosphate dehydrogenase
H04	NM_000194	HPRT1	Hypoxanthine phosphoribosyltransferase 1
H05	NM_001002	RPLP0	Ribosomal protein, large, P0
H06	SA_00105	HGDC	Human Genomic DNA Contamination
H07	SA_00104	RTC	Reverse Transcription Control
H08	SA_00104	RTC	Reverse Transcription Control
H09	SA_00104	RTC	Reverse Transcription Control
H10	SA_00103	PPC	Positive PCR Control
H11	SA_00103	PPC	Positive PCR Control
H12	SA_00103	PPC	Positive PCR Control

Table S4. Fold-Change (2^(-Delta Delta CT)) is the normalized gene expression (2^(- Delta CT)) in the Test Sample divided the normalized gene expression (2^(- Delta CT)) in the Control Sample of PNT2 cells. Fold-Regulation represents fold-change results in a biologically meaningful way. Fold-change values greater than one indicates a positive- or an up-regulation, and the fold-regulation is equal to the fold-change. Fold-change values less than one indicate a negative or down-regulation, and the fold-regulation is the negative inverse of the fold-change.

Position	Gene Symbol	Fold Regulation
A01	CCL11	4064.11
A02	CCL13	1789.40
A03	CCL16	471.98
A04	CCL19	108.45
A05	CCL2	56.38
A06	CCL3	4570.86
A07	CCL5	321.29
A08	CCL7	3132.83
A09	CCL8	1537.75
A10	CCR1	163.07
A11	CCR2	5728.88
A12	CCR3	10.22
B01	CCR5	7782.28
B02	CD1A	2337.96
B03	CD1B	453.15
B04	CD1C	141.42
B05	CD1D	2998.34
B06	CD2	6984.02
B07	CD209	6205.05
B08	CD28	471.98
B09	CD4	1379.97
B11	CD40LG	20122.45
B12	CD44	8.29
C02	CD80	1366.18
C03	CD86	471.98
C04	CD8A	458.36
C05	CDC42	33.76
C06	CDKN1A	10.01
C07	CEBPA	7.54
C08	CLEC4C	407.39
C09	CSF1R	129.32
C11	CXCL1	157.40
C12	CXCL10	1358.25
D01	CXCL12	2741.42
D02	CXCL2	21.41
D03	CXCR1	471.98
D04	CXCR4	153330.96
D06	FAS	25.43
D07	FCER1A	471.98
D08	FCER2	471.98
D09	FCGR1A	537.75
D10	FLT3	471.98
E02	HLA-DPA1	82.58
E03	ICAM1	12.51
E04	ICAM2	15.64
E05	IFNG	1129.25
E06	IL10	156.22
E07	IL12A	165.36
E08	IL12B	1167.60
E09	IL16	111.92
E10	IL2	3444.00
E11	IL6	1505.44
E12	CXCL8	147.79
F02	IRF8	2.39
F03	ITGAM	180.18
F04	ITGB2	2.72
F06	LYN	2.74
F07	MIF	3.30
F09	PTPRC	17238.12
F10	RAC1	23.70
F11	RELA	2.29
F12	RELB	8.91
G01	STAT3	4.98
G04	TGFB1	3.60
G05	THBS1	23.30
G06	TLR1	6.63
G08	TLR7	641.54
G09	TLR9	58.68
G10	TNF	7.36
G11	TNFSF11	191.88
G12	VCAM1	4336.80
H02	B2M	8.66
H04	HPRT1	8.94
H05	RPLP0	2.41
B10	CD40	-2.25
D12	HLA-A	-7.21
E01	HLA-DMA	-2.84
F01	IRF7	-7.18
F05	LRP1	-14.98
G02	TAP2	-8.10
G03	TAPBP	-24.16
H01	ACTB	-17.07
H03	GAPDH	-10.92
